# Diarrhea as a cause of mortality in a mouse model of infectious colitis

**DOI:** 10.1186/gb-2008-9-8-r122

**Published:** 2008-08-04

**Authors:** Diana Borenshtein, Rebecca C Fry, Elizabeth B Groff, Prashant R Nambiar, Vincent J Carey, James G Fox, David B Schauer

**Affiliations:** 1 Department of Biological Engineering, Massachusetts Institute of Technology, Massachusetts Avenue, Cambridge, MA 02139, USA; 2 Center of Environmental Health Sciences, Massachusetts Institute of Technology, Massachusetts Avenue, Cambridge, MA 02139, USA; 3 Division of Comparative Medicine, Massachusetts Institute of Technology, Massachusetts Avenue, Cambridge, MA 02139, USA; 4 Harvard Medical School, Longwood Avenue, Boston, MA 02115, USA; 5 Current address: Department of Environmental Sciences and Engineering, The University of North Carolina at Chapel Hill, Dauer Drive, Chapel Hill, NC 27599, USA; 6 Current address: Genzyme Corporation, Mountain Road, Framingham, MA 01701, USA

## Abstract

**Background:**

Comparative characterization of genome-wide transcriptional changes during infection can help elucidate the mechanisms underlying host susceptibility. In this study, transcriptional profiling of the mouse colon was carried out in two cognate lines of mice that differ in their response to *Citrobacter rodentium *infection; susceptible inbred FVB/N and resistant outbred Swiss Webster mice. Gene expression in the distal colon was determined prior to infection, and at four and nine days post-inoculation using a whole mouse genome Affymetrix array.

**Results:**

Computational analysis identified 462 probe sets more than 2-fold differentially expressed between uninoculated resistant and susceptible mice. In response to *C. rodentium *infection, 5,123 probe sets were differentially expressed in one or both lines of mice. Microarray data were validated by quantitative real-time RT-PCR for 35 selected genes and were found to have a 94% concordance rate. Transcripts represented by 1,547 probe sets were differentially expressed between susceptible and resistant mice regardless of infection status, a host effect. Genes associated with transport were over-represented to a greater extent than even immune response-related genes. Electrolyte analysis revealed reduction in serum levels of chloride and sodium in susceptible animals.

**Conclusion:**

The results support the hypothesis that mortality in *C. rodentium*-infected susceptible mice is associated with impaired intestinal ion transport and development of fatal fluid loss and dehydration. These studies contribute to our understanding of the pathogenesis of *C. rodentium *and suggest novel strategies for the prevention and treatment of diarrhea associated with intestinal bacterial infections.

## Abstract 2

Analysis of gene expression in the colons of *Citrobacter rodentium*-infected susceptible and resistant mice suggests that mortality is associated with impaired intestinal ion transport.

## Background

Acute diarrheal illness is one of the most important health problems in the world today, particularly in young children in developing countries. This life-threatening illness occurs in approximately four billion individuals per year and causes more than two million deaths worldwide each year [1]. The most common cause of diarrhea is gastrointestinal infection. Infection results in increased intestinal secretion and/or decreased intestinal absorption followed by fluid and electrolyte loss and dehydration that can be fatal if not treated [2,3]. Among the most important bacterial causes of diarrhea are enteropathogenic and enterohaemorrhagic *Escherichia coli *(EPEC and EHEC, respectively) [4]. These pathogens produce ultrastructural changes characterized by intimate bacterial adhesion to the apical surface of enterocytes, effacement of microvilli, and pedestal formation, which are called 'attaching and effacing' (A/E) lesions. The pathophysiology of diarrhea due to infection with A/E pathogens is not well understood. Proposed mechanisms include decreased absorptive surface epithelium, disruption of tight junctions and intestinal barrier function, impaired ion transport, and induction of inflammation [5,6].

*Citrobacter rodentium*, a murine A/E pathogen, possesses similar virulence factors as EPEC and EHEC, and produces comparable ultrastructural changes in the distal colon of infected mice (reviewed in [7,8]). Typically, this organism causes severe, but self-limiting, epithelial hyperplasia with a variable degree of inflammation in the distal colon of most inbred and outbred lines of laboratory mice. Exceptions include suckling animals or C3H substrains (independent of toll-like receptor 4 status), which demonstrate 60-100% mortality by approximately two weeks after infection with *C. rodentium *[9-12]. We recently discovered that adult FVB/N mice (FVB) are also extremely susceptible to *C. rodentium *infection [13]. Inbred FVB mice are derived from outbred Swiss Webster (SW) mice and, since SW are known to be resistant, comparative studies between these cognate lines of mice were performed. Twelve-week old FVB mice infected with *C. rodentium *developed a high degree of mortality and severe colitis compared with their outbred SW counterparts, which had more typical subclinical disease in response to infection. Differences in disease outcome were observed despite comparable expression of tumor necrosis factor-α, interferon-γ, and inducible nitric oxide synthase in susceptible and resistant animals. The results of our previous study suggested that the cause of death in *C. rodentium*-infected FVB mice was hypovolemia due to dehydration [13]. To characterize the mechanistic basis for the striking difference in disease outcome between two closely related lines of mice, we used microarray analysis to determine global patterns of gene expression in susceptible FVB and resistant SW mice infected with *C. rodentium*. GeneChips^® ^from Affymetrix were employed to identify and quantify both host-dependent and infection-dependent alterations in host gene expression; results were confirmed by quantitative real-time PCR (qRT-PCR), immunohistochemistry, and serology. We identified predominant functional categories of differentially regulated genes and potential candidates for susceptibility, both of which have implications for future studies of *C. rodentium *pathogenesis. Based on these findings, we propose testable hypotheses about newly implicated host genes and their potential role in the development of infectious colitis and diarrhea.

## Results

### Infection of FVB and SW mice with *C. rodentium*

To characterize the differences in gene expression between susceptible FVB and resistant SW mice, animals were analyzed before *C. rodentium *infection and at two different time points post-inoculation. Time points were selected to reveal differentially expressed genes prior to infection (uninoculated), following establishment of infection but before the development of disease (4 days post-inoculation (dpi)), and after the development of colitis but before the development of appreciable mortality (9 dpi). As expected, sham-dosed 12-week old mice were found to be indistinguishable at 4 and 9 dpi; therefore, samples from these uninoculated control animals were combined and treated as a single group for each line of mouse (experimental design is presented in Additional data file 1).

**Figure 1:**
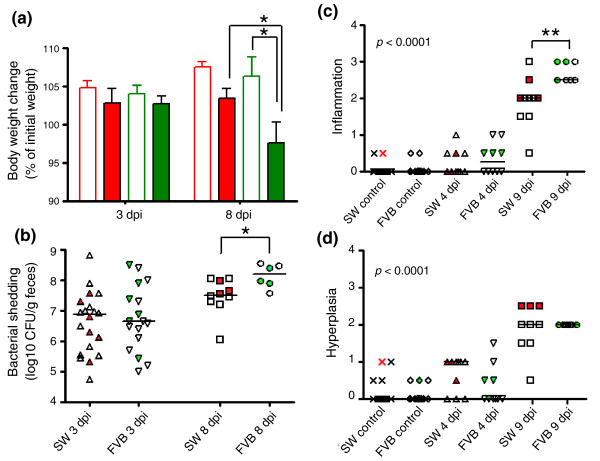
*C. rodentium *infection in adult susceptible inbred FVB mice and resistant outbred SW mice. **(a) **Significant weight loss was observed in infected FVB mice at 8 dpi (*p *< 0.05). Weight was normalized and expressed as percent change of initial baseline. Red and green indicate SW and FVB mice, respectively; open and filled bars represent uninoculated and infected mice, respectively. Values are mean ± standard error of the mean. **(b) **Fecal bacterial counts were similar in both lines of mice at 3 dpi, but FVB mice had higher bacterial shedding at 8 dpi (*p *< 0.05). Bacterial counts were log10 transformed. **(c) **FVB mice infected with *C. rodentium *developed colonic inflammation that was significantly more severe than the milder colitis in SW mice at 9 dpi (*p *< 0.0001). **(d) **Infected FVB and SW mice developed comparable hyperplasia at 9 dpi. Experimental groups included 20, 10, and 7 uninoculated control, 4 dpi, and 9 dpi FVB mice, respectively, and 16, 10, and 10 SW mice in the corresponding groups. Each symbol represents one animal; filled symbols in red or green represent SW or FVB mice selected for array analysis. Mean or median lines for each group are presented. *p < 0.05; **p < 0.01.

Details of FVB susceptibility to *C. rodentium *infection were previously reported [13]. Here, FVB and SW mice infected with *C. rodentium *developed comparable alterations in body weight, fecal bacterial shedding, and no appreciable colonic lesions at 3-4 dpi (Figure 1). By 8 dpi, body weight gain was not significantly different between infected and uninoculated control SW mice (107.5 ± 2.0% and 106.3 ± 1.8% of initial body weight, respectively; Figure 1a), whereas infected FVB mice developed significant weight loss compared to uninoculated controls (97.6 ± 2.2% and 103.4 ± 1.8%, respectively, *p *< 0.05). Likewise, fecal bacterial shedding was higher in FVB mice than in SW mice at 8 dpi (8.1 ± 0.2 versus 7.5 ± 0.2 log10 CFU/g feces, respectively, *p *< 0.05; Figure 1b). At 9 dpi, FVB mice infected with *C. rodentium *had significant pathological lesions, including colonic inflammation and hyperplasia (Figure 1c,d), and mild dysplasia (data not shown). Infected SW mice developed comparable hyperplasia, but less inflammation and no dysplasia at 9 dpi (*p *< 0.0001). The median lesion scores for infected versus control FVB mice were 2.5 versus 0 for inflammation, 2 versus 0 for hyperplasia, and 0.5 versus 0 for dysplasia. The median lesion scores for infected versus control SW mice were 2 versus 0 for inflammation, 2 versus 0 for hyperplasia, and 0 versus 0 for dysplasia. Samples for microarray analysis were selected based on the clinical signs, infection status, and severity of lesions, and are shown in Figure 1.

### Gene expression analysis of FVB and SW mice during *C. rodentium *infection

**Figure 2:**
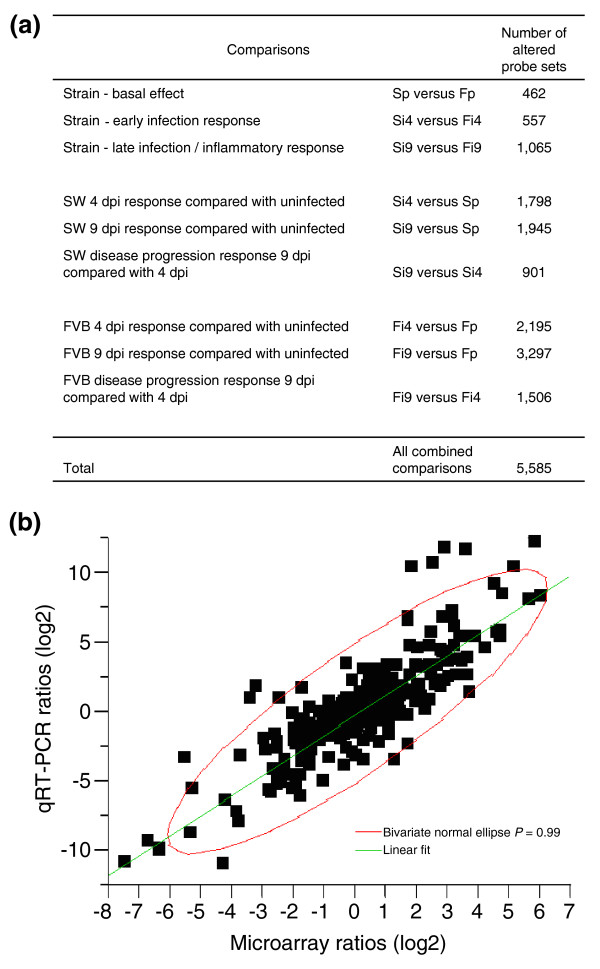
Differential expression of genes between and within the lines of mice prior to, and in response to, *C. rodentium *infection. **(a) **Summary of transcripts differentially expressed in individual and combined comparisons. The analysis was performed using an Affymetrix whole mouse genome oligonucleotide chip (430 2.0 Array), which contains >45,000 probe sets comprising expression levels of >39,000 transcripts and variants from >34,000 well-characterized mouse genes. The normalization and processing of the results were performed using DNA-Chip Analyzer (dChip) software implementing model-based expression analysis. One percent of the total probe sets presented on the array were more than two-fold differentially expressed between SW and FVB mice prior to infection. In response to *C. rodentium *inoculation, 11.4% of the probe sets were either induced or repressed in one or both of the lines of mice. There were more differentially expressed genes in response to infection in susceptible FVB mice than in resistant SW mice, especially as disease progressed. Overall, alterations in 12.4% of the probe sets were detected throughout the experiment. **(b) **Validation of microarray results by qRT-PCR (TaqMan) of selected genes. Transcript levels were normalized to the endogenous control GAPDH, and expressed as fold change compared with untreated control FVB mice, which were set at 1, using the Comparative Ct method. The resultant log2 ratios were matched with corresponding log2 ratios detected in microarray analysis and subjected to Pearson correlation analysis. Significant correlation was observed between the two assays (Pearson correlation coefficient r = 0.87, R^2 ^= 0.75, *p *< 0.0001). Pearson correlations for individual genes ranged from 0.67 to 1. Only two out of 35 examined genes did not confirm the array results, yielding a predictability rate of 94%.

Transcriptional profiling was performed on RNA isolated from full-thickness descending colon tissues. Differential expression analysis of pairwise comparisons (see Material and methods) identified 462 probe sets (1% of the total number of probe sets) significantly different between SW and FVB mice prior to infection (Figure 2a). In response to *C. rodentium *inoculation, 5,123 probe sets (11.4%) were either induced or repressed by more than two-fold in one or both of the lines of mice. The number of significantly modulated genes in response to infection was greater in susceptible FVB mice than in resistant SW mice, particularly as disease progressed. Specifically, infected FVB mice had 2,195 and 3,297 differentially expressed probe sets at 4 and 9 dpi, respectively, compared with uninoculated controls, whereas infected SW mice had 1,798 and 1,945 differentially expressed probe sets at 4 and 9 dpi, respectively, compared to uninoculated controls (Figure 2a). Overall, alterations in 5,585 (12.4%) probe sets were detected during the course of the experiment. Most of the differences were within a ±7-fold range (Additional data file 2).

### Validation of microarray results by qRT-PCR

To confirm the results obtained with GeneChips^®^, quantitative real-time fluorigenic RT-PCR (TaqMan) was performed for 35 selected genes. Correlation analysis was performed by comparing expression ratios from microarray results versus ratios determined by TaqMan analysis (Figure 2b). A significant correlation was observed between the two assays (Pearson correlation coefficient r = 0.87, R^2 ^= 0.75, *p *< 0.0001). Individual Pearson correlation coefficients ranged from 0.67 to 1 in all but 2 out of 35 genes (*Crry *and *Slc10a2*; Additional data file 3). The overall concordance of the microarray results with qRT-PCR was 94%, which compares favorably or even exceeds that reported for data processing by dChip [14]. Side-by-side comparisons of microarray and qRT-PCR results are presented in Additional data file 4.

### Analysis of genes differentially expressed between susceptible and resistant mice (host effect)

**Figure 3:**
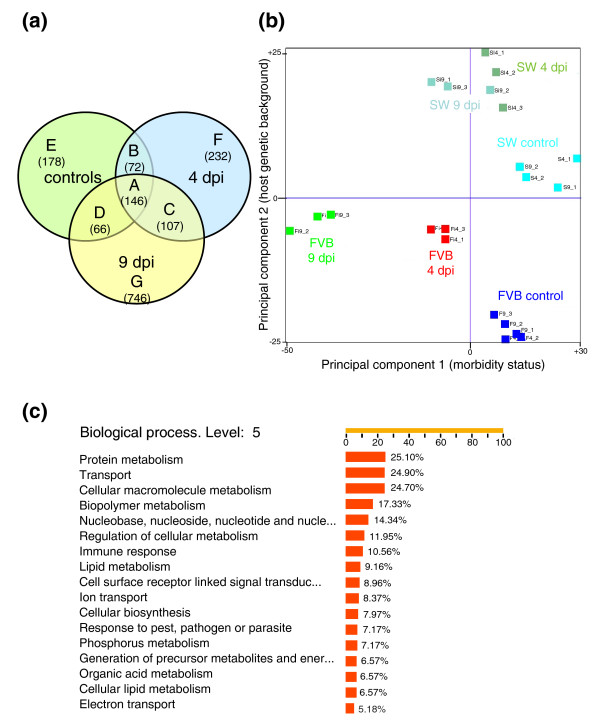
Genes contributing to host susceptibility. **(a) **Comparative analysis of gene expression profiles of SW versus FVB genes prior to infection or at 4 and 9 dpi is shown as a Venn diagram. Overall, 1,547 genes were differentially expressed between the lines of mice (Additional data file 5) and divided into 7 distinct subsets. Group A represents genes that were differentially expressed between the mouse lines at all time points. Groups B, C, and D represent genes that were differentially expressed at two conditions/time points. Groups E, F, and G represent genes unique to uninfected status, 4 dpi and 9 dpi, respectively. Each subset represents the comparison of resistant outbred SW mice to susceptible inbred FVB mice at the indicated time point. Numbers in parentheses represent the number of differentially expressed probe sets in each group. Significantly enriched GO clusters (*p *< 0.05 by hypergeometric test) for each group and for all sets of genes with host effect are given in Additional data file 20. **(b) **PCA distinguished SW from FVB mice in PC2. PC1 established negative correlation of infected and uninoculated FVB mice, but did not discriminate SW mice by infection status. Thus, PC1 represents morbidity associated with infection. **(c) **The prevalence of genes within GO categories was assessed by FatiGO analysis. Only categories containing more than 5% of genes are shown. Genes from transport processes were overrepresented.

To identify genes that were differentially expressed between susceptible FVB mice and resistant SW mice as a function of time during infection, comparative analysis of common and unique genes modulated at individual time points was performed (Sp versus Fp or Si4 versus Fi4 or Si9 versus Fi9; see the 'Array design and hybridization' section in Materials and methods for descriptions of the different groups). The results presented in the Venn diagram in Figure 3a represent seven subsets of differentially expressed genes between SW and FVB mice. Overall, 1,547 probe sets (3.4%), were more than two-fold differentially expressed between the two lines of mice (a complete list of genes with host effect is presented in Additional data file 5).

This set of genes was subjected to principal component analysis (PCA; Figure 3b; Additional data file 6), yielding robust separation of SW and FVB mice for all time points in principal component (PC)2. Consistent with their inbred strain background, there was tighter clustering of uninoculated control FVB mice than uninoculated control outbred SW mice. PC1 yielded robust separation of infected from uninoculated control FVB mice, but was not able to discriminate infected from uninoculated control SW mice. Thus, PC1 is composed of factors contributing to morbidity associated with infection. As expected, similar results were obtained by hierarchical clustering (Additional data file 7). Distinct branches for uninoculated, 4 dpi, and 9 dpi FVB mice, along with robust separation between uninoculated and infected SW mice, was in good agreement with the results of PCA. Interestingly, PCA applied on any of the individual subgroups presented in the Venn diagram was not sufficient to clearly distinguish between experimental groups (data not shown). This suggested that all 1,547 genes were required for reliable discrimination of mice by host genetic background and infection status and, hence, were called 'genes with host effect'.

To characterize these transcripts biologically, enrichment analysis of genes with host effect by their functional annotation with Gene Ontology (GO) was performed. Approximately 25% of these genes were assigned to the GO category 'transport', making it one of the most prevalent categories. On the other hand, only 11% of genes were assigned to the 'immune response' category (Figure 3c). Similar results were obtained when the most significantly differentially expressed genes with host effect were analyzed (more than eight-fold difference, presented in Additional data files 8 and 9), which identified 'transporter activity' among the most significantly enriched functional categories; using the hypergeometric test for establishing a cutoff threshold revealed significant enrichment (*p *< 0.05; Additional data file 10).

To identify host-dependent temporal changes upon infection, an analysis was used that contrasts the magnitude of gene expression induced upon infection in one line of mouse (ratio relative to uninoculated) to changes induced upon infection in the other line of mouse (ratio relative to uninoculated), termed delta eta (Materials and methods). Out of 1,385 probe sets detected by delta eta analysis (Additional data file 11), 468 were differentially expressed between the lines of mice at 4 dpi, 1,173 probe sets at 9 dpi, and 256 at both time points. The most significant candidates differentially expressed by more than 8-fold included 36 genes, the majority of which were also identified by pairwise comparisons described above. Interestingly, delta eta analysis also discovered novel candidates that were not identified by pairwise comparisons (Additional data files 11 and 12), including the gene for aquaporin 4 (*Aqp4*), which was upregulated in SW mice but not in FVB mice. Functional classification of these transcripts revealed significant enrichment in 'transporter activity', 'immune response', 'antigen binding', 'channel or pore class transporter activity',' and 'carbohydrate binding' categories (*p *< 0.05; Additional data file 13).

To ensure that the results were not biased by using a single computational technique, we also analyzed these data using a Robust Multichip Average algorithm and linear modeling with a moderate *t*-test (see Materials and methods; Additional data files 14-18). These results also identified significant enrichment of GO categories with transport functions among genes altered by infection in a host-dependent manner (*p *< 0.0005; Additional data files 16 and 17).

### Differential expression of genes involved in intestinal ion transport and its regulation

**Figure 4:**
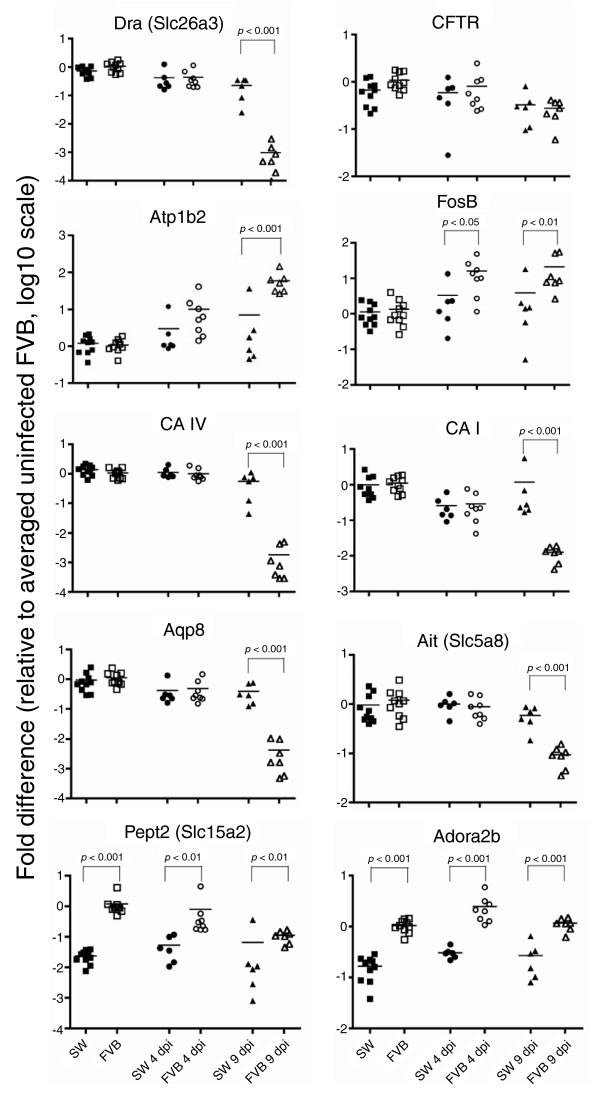
qRT-PCR of genes involved in intestinal transport and its regulation. The expression of genes was normalized to uninoculated FVB mice. Each symbol represents one animal. Lines indicate group means.

The prevalence of transport genes within the set of differentially expressed transcripts detected by different analytical methods supports the hypothesis that high mortality in *C. rodentium*-infected FVB mice results from severe diarrhea and dehydration as a consequence of electrolyte imbalance [13]. We next concentrated on genes implicated in intestinal ion transport as well as genes with regulatory and/or signaling functions. GO annotations are not complete for all transcripts, and the genes involved in intestinal transport do not comprise a single distinct group in the pathway analysis. Therefore, differentially expressed genes (Table 1; Figure 4; Additional data file 19) were selected for validation by qRT-PCR and further characterization based on our current understanding of colonic ion transport (reviewed in [15,16]). Four general patterns of gene expression changes were observed.

**Table 1:** Genes involved in intestinal ion transport and its regulation

Probe set ID	Gene	Gene name/aliases	Locus link	SW over FVB, control*	SW over FVB, 4 dpi	SW over FVB, 9 dpi	Main functions
**Transporters**							
1425382_a_at 1434449_at 1447745_at	*aquaporin 4*	*Aqp4*, *mMIWC*	11829	-1.88		1.15	Water transport
1417828_at	***aquaporin 8***	*Aqp8*	11833			3.14^†^	Water transport
1449475_at	*ATPase, H*^+^/*K*^+^*transporting, nongastric, alpha polypeptide*	*cHKA*, *Atp12a*	192113			1.68	Potassium and proton ion transport
1422009_at 1435148_at	***ATPase, Na***^+^ ***/K***^+^ ***transporting, beta 2 polypeptide***	*Atp1b2*, *Amog*	11932		-2.99^†^	-2.56	Potassium and sodium ion transport
1435945_a_at	*potassium intermediate/small conductance calcium-activated channel, subfamily N, member 4*	*Kcnn4*, *SK4*, *IK1*	16534			-0.93^†^	Potassium ion transport
1425088_at	*sodium channel, nonvoltage-gated, type I, alpha*	*mENaC*, *Scnn1a*	20276			0.9	Sodium ion transport
1417623_at 1448780_at	*solute carrier family 12, member 2*	*Nkcc1*, *Slc12a2*	20496			-0.96^†^	Sodium:potassium: chloride cotransport
1417600_at	***solute carrier family 15 (H***^+^ ***/peptide transporter), member 2***	*Slc15a2*, *Pept2*	57738	-5.28^‡^	-3.69^§^	-5.53^‡^	Oligopeptide and proton transport
1419343_at	*solute carrier family 15 (oligopeptide transporter), member 1*	*Slc15a1*, *Pept1*	56643		0.59	3.79^†^	Oligopeptide and proton transport
1429467_s_at 1421445_at 1427547_a_at	***solute carrier family 26, member 3***	*Slc26a3*, *Dra*	13487			6.04^§^	Anion exchanger activity, transport
1425606_at	***solute carrier family 5 (iodide transporter), member 8***	*Ait*, *Slc5a8*	216225			1.79^†^	Ion transport
1437259_at	*solute carrier family 9 (sodium/hydrogen exchanger), member 2*	*NHE2*, *Slc9a2*	226999			2.12^§^	Sodium transport
1441236_at	*solute carrier family 9 (sodium/hydrogen exchanger), member 3*	*NHE3*, *Slc9a3*	105243			0.84	Sodium transport
							
**Regulators**							
1434430_s_at 1434431_x_at 1450214_at	***adenosine A2b receptor***	*Adora2b*	11541	-1.72^¶^	-2.89^§^	-1.89^†^	G-protein coupled receptor protein signaling pathway
1431130_at	*calcineurin B homologous protein 2 (2010110P09Rik)*	*Chp2*, *Cbhp2*	70261			2.38^§^	Sodium ion transport; regulation of pH
1455869_at	*calcium/calmodulin-dependent protein kinase II, beta*	*Camk2b*	12323	-0.85	-3.39	-2.46	G1/S transition; calcium transport and signaling
1416193_at	***carbonic anhydrase 1***	*Car1*, *CA I*	12346			3.4^†^	One-carbon compound metabolism, maintenance of pH
1448949_at 1418094_s_at	***carbonic anhydrase 4***	*Car4*, *CA IV*	12351			5.32^†^	One-carbon compound metabolism, maintenance of pH, anion transport
1422134_at	***FBJ osteosarcoma oncogene B***	*Fosb*	14282		-2.54^†^	-1.75	Regulation of transcription
1435162_at	*protein kinase, cGMP-dependent, type II*	*Prkg2*	19092			-1.69^†^	Signal transduction
1438115_a_at 1438116_x_at 1450982_at	*solute carrier family 9 (sodium/hydrogen exchanger), isoform 3 regulator 1*	*NHERF1*, *EBP50*, *Slc9a3r1*	26941			1.1^†^	Regulation of sodium: hydrogen exchange
1451602_at	*sorting nexin 6*	*Snx6*, *TFAF2*	72183	-3.98^‡^	-4.01	-4.95^§^	Protein and ion transport

*The numbers represent log2 ratios resulting from individual groups comparison. Significance by *t*-test was: ^†^*p *< 0.05; ^§^*p *≤ 0.005; ^¶^*p *≤ 0.0005; ^‡^*p *≤ 0.00005. Genes whose expression was confirmed by qRT-PCR are in bold.

First, a number of transcripts had distinct transcriptional activity between the two lines of mice at all time points. For example, FVB mice had consistently four- to eight-fold higher expression of the adenosine A2B receptor gene (*Adora2b*).

Second, a group of genes, although consistently overexpressed in FVB mice compared to SW mice, also exhibited different expression as a function of time during infection. The Sorting nexin gene (*Snx6*; overexpressed in FVB mice by 16- to 31-fold compared with SW mice) had increased expression at 4 dpi by approximately 2-fold in both lines of mice. However, at 9 dpi, expression of *Snx6 *remained elevated in FVB mice, but returned to normal in SW mice. Another example was proton-dependent high affinity oligopeptide transporter *Pept2 *(*Slc15a2*), which was overexpressed in FVB mice by 15- to 51-fold. *Slc15a2 *was upregulated in infected SW mice by 2-fold at 4 dpi and downregulated by 4-fold at 9 dpi, whereas in infected FVB mice its expression decreased by 11-fold at 9 dpi.

Third, some genes were differentially expressed in infected mice as early as 4 dpi, indicating a rapid response and/or involvement in regulation. For example, expression of the Na^+^/K^+^-ATPase beta 2 subunit gene (*Atp1b2*) was increased in SW mice by only 2.5- and 6-fold at 4 and 9 dpi, whereas in infected FVB mice it was induced by 10- and 55-fold, respectively. Similar changes were observed in the transcription factor FBJ osteosarcoma oncogene B gene (*Fosb*), with 3-fold increased expression in infected SW mice at both time points, and 12- and 16-fold changes in FVB mice at 4 and 9 dpi, respectively. The calcium/calmodulin-dependent protein kinase gene (*Camk2b*) had 4-fold decreased expression in SW mice at 4 dpi, but 2.5-fold increase in expression in FVB mice at 9 dpi. Expression of the basolateral water channel aquaporin gene (*Aqp4*) was induced in both lines of mice at 4 dpi, but more significantly in SW mice (approximately seven-fold increase compared with approximately two-fold increase in FVB mice). At 9 dpi, expression of *Aqp4 *remained elevated in SW mice, but returned to baseline in FVB mice (Table 1; Additional data file 2).

The fourth and largest group was composed of genes differentially expressed between infected FVB and SW mice as disease progressed, at 9 dpi. Many of these genes had remarkable decreases in expression, including down-regulated in adenoma *Dra *(*Slc26a3*; 1,100- versus 3-fold change in FVB versus SW mice at 9 dpi), aquaporin *Aqp8 *(268- versus 2-fold change), and carbonic anhydrases *CA I *and *CA IV *(87- versus 0.8-fold, and 586- versus 2.5-fold change, respectively). Less dramatic changes included downregulation of the sodium/hydrogen exchangers *Slc9a2 *(*NHE2*; 11- versus 2.5-fold decrease in FVB versus SW mice at 9 dpi) and *Slc9a3 *(*NHE3*; 8- versus 3-fold change), the apical iodide transporter (*Slc5a8*; 13- versus 1.6-fold change), the epithelial Na+ channel (ENaC) alpha subunit encoded by *Scnn1a *(2.7-fold decrease in FVB mice versus no change in SW mice), the sodium-hydrogen exchanger regulatory factor *Slc9a3r1 *(*NHERF1 *a.k.a. *EBP-50*; 2-fold versus no change) and *2010110P09Rik *encoding the calcineurin B homologous protein Chp2 (8- versus 2-fold change). Expression of the ouabain-sensitive H^+^,K^+^-ATPase *Atp12a *(*cHKA*) had decreased in FVB mice by 2.5-fold but increased in SW mice by 1.5-fold at 9 dpi. Likewise, the potassium channel *Kcnn4 *(*SK4*) and the cGMP-dependent protein kinase *Prkg2 *were upregulated by more than 2-fold in infected FVB mice without notable changes in the expression of these genes in SW mice (Table 1; Additional data file 19).

In addition to genes identified by microarray analysis, we verified the expression of cystic fibrosis transmembrane conductance regulator homolog (*Cftr*), which serves as the main chloride channel in the intestine and other tissues. Two transcripts corresponding to this gene showed opposite results by microarray analysis (Additional data files 2 and 19), bringing into question the importance of changes in expression of this gene in our model. Nevertheless, to create a clearer picture of intestinal ion transport in *C. rodentium*-infected mice, we analyzed expression by qRT-PCR and found no difference in *Cftr *expression between SW and FVB mice, though a subtle (4-fold) decrease in mRNA levels was observed in FVB mice at 9 dpi (Figure 4).

### Expression of *Dra *and *CA IV *gene products

**Figure 5:**
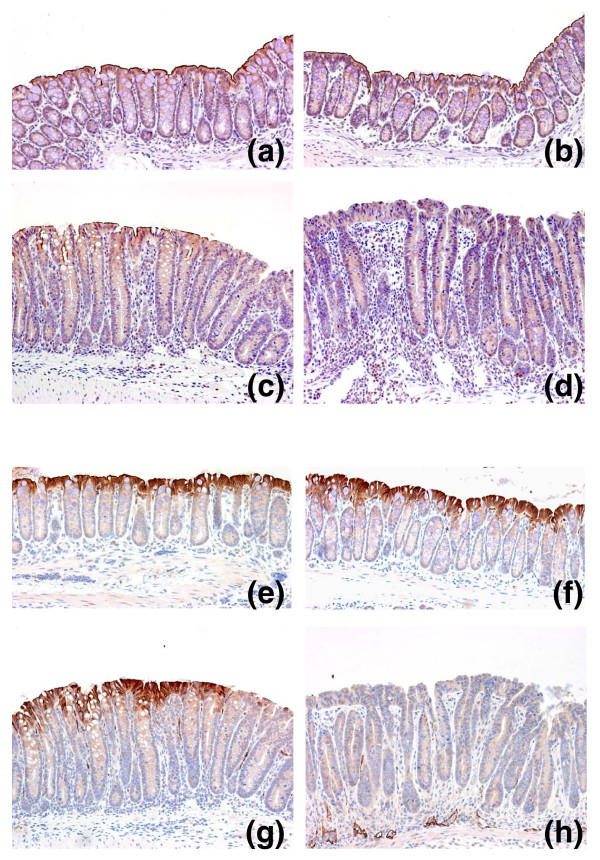
Validation of Dra and CA IV expression by immunohistochemistry. Colonic samples were stained with antibodies against **(a-d) **Dra or **(e-h) **CA IV. Normal apical expression of proteins was observed in distal colon from uninoculated SW (a,e) and FVB (b,f) mice. By 9 dpi, partial loss of protein expression was observed in infected SW mice (c,g) compared with complete lack of expression in infected FVB mice (d,h). Original magnifications are 200×.

To validate the results of genomic profiling at the transcriptional level, we analyzed expression of the most significantly downregulated proteins, Dra and CA IV, by immunohistochemistry (Figure 5). Strong apical expression of Dra was observed throughout the colon in uninoculated SW and FVB mice (Figure 5a,b), as has been reported previously [17]. By 9 dpi, patchy loss of Dra expression with detectible signal in the adjacent segments of epithelium was found in some areas of the distal colon in SW mice (Figure 5c). Infected FVB mice, on the other hand, demonstrated complete lack of Dra expression in the distal colon (Figure 5d). Dra exhibited a gradient of expression from the distal to proximal colon, with levels of expression in the proximal colon of infected FVB mice approximating those in the distal colon of uninoculated control FVB mice (data not shown). Similar results were found for CA IV. The expression of CA IV in uninoculated SW and FVB mice was localized to the surface epithelium, as has been reported previously [18] (Figure 5e,f). There were diffuse areas with partial loss of CA IV staining in infected SW mice (Figure 5g) compared with complete lack of CA IV expression in the distal colon of FVB mice at 9 dpi (Figure 5h). No signal was detected using normal IgG as a negative control.

### Alterations in serum electrolytes

**Figure 6:**
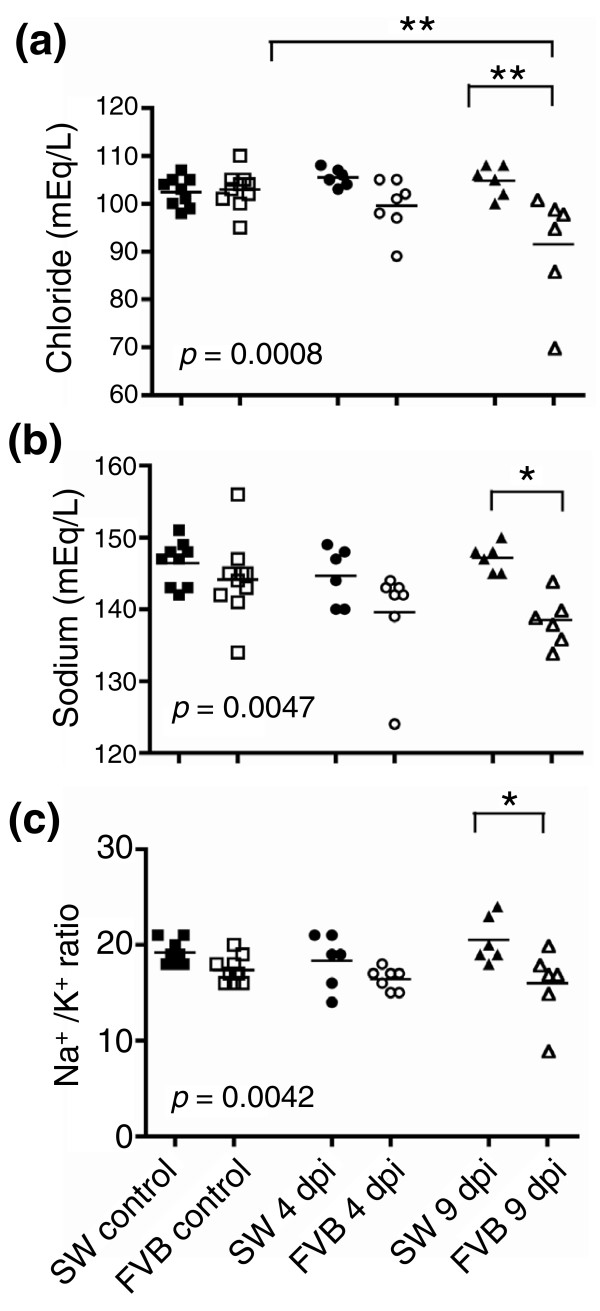
Serum electrolyte levels. Infected FVB mice had **(a) **hypochloremia, **(b) **hyponatremia, and **(c) **altered Na^+^/K^+ ^ratio in serum compared with infected SW mice. Each symbol represents an individual mouse; lines indicate means of the group. *p < 0.05; **p < 0.01.

Gene expression profiling identified significant differences in expression of ion transporters that could contribute to diarrhea and fluid and electrolyte loss in FVB mice. Because severe alterations in electrolyte homeostasis can lead to changes in serum chemistry, we measured serum electrolytes in SW and FVB mice (Figure 6). While no changes in electrolyte levels were detected in SW mice during infection, infected FVB mice developed significant hypochloremia and hyponatremia (*p *< 0.001). The mean concentrations of serum chloride were 102.4 ± 1.8, 105.5 ± 2.3, and 104.8 ± 2.3 mEq/l in SW mice before infection and at 4 and 9 dpi, respectively, and 102.9 ± 1.8, 99.6 ± 2.1, and 91.5 ± 2.3 mEq/l in FVB mice before infection and at 4 and 9 dpi, respectively. Sodium concentrations in serum were 146.4 ± 1.5, 144.7 ± 1.9, and 147.2 ± 1.9 mEq/l in SW mice before infection and at 4 and 9 dpi, respectively, and 144.2 ± 1.5, 139.6 ± 1.7, and 138.5 ± 1.9 mEq/l in FVB mice before infection and at 4 and 9 dpi, respectively. Anion gap, total CO_2 _and potassium levels were comparable in all groups at all time points (data not shown), whereas Na^+^/K^+ ^ratios were lower in infected FVB mice at 9 dpi (16.0 ± 0.9 compared with 20.5 ± 0.9 in SW at 9 dpi, *p *< 0.005).

## Discussion

Global expression profiling is emerging as a powerful method to characterize host-pathogen interactions [19], although many of the studies to date have been performed on cultured cells rather than *in vivo*. Here we present the first global expression analysis of host response to infection with an A/E bacterial pathogen. The goal of this study was to characterize the pathophysiological basis of susceptibility to *C. rodentium *infection in FVB mice. In order to accomplish this, we performed quantitative analysis of the transcriptome of distal colon tissues from FVB and SW mice before and during *C. rodentium *infection. These studies revealed profound basal and infection-dependent differences between susceptible FVB mice and resistant SW mice.

Many studies of *C. rodentium *interactions with host cells have focused on immune responses and the actin cytoskeleton [7,8]. Indeed, a substantial fraction of functional categories of differentially expressed genes that we identified are involved in immune responses and cellular adhesion (Additional data file 20). However, results from our group as well as others suggest that innate and adaptive immunity, though important in determining the development of morbidity, is not the most critical factor in determining mortality. Susceptible FVB mice are able to clear *C. rodentium *infection, are fully protected against mortality by fluid therapy (without affecting clinical disease and the severity of colonic lesions), and demonstrate similar expression of pro-inflammatory and immunomodulatory genes in the colon compared to resistant SW mice [13]. Likewise, the status of LPS responsiveness (toll-like receptor 4 sufficiency) in different substrains of C3H mice infected with a high number of *C. rodentium *does not affect the incidence of mortality [11,12]. These observations suggest that the immune status of mice does not contribute to the ability to survive infection, but most likely affects clearance of the pathogen.

**Figure 7:**
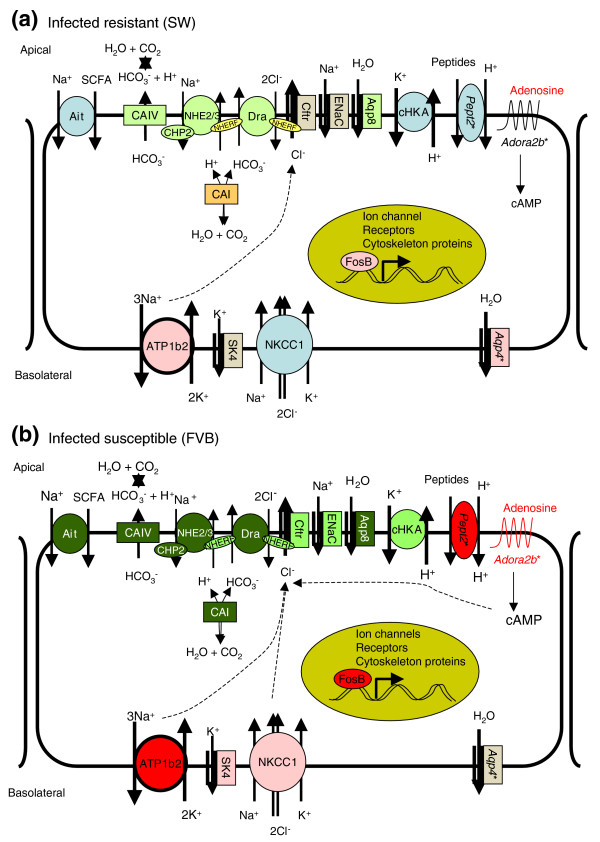
Working model for the pathogenesis of colonic ion transport in fatal diarrhea in *C. rodentium*-infected FVB mice. Normal ion transport in the large intestine is mediated largely by the coupled action of the anion exchanger DRA, CFTR, sodium/proton exchangers NHE2/3, potassium transporters and carbonic anhydrases (see Discussion). **(a) **alterations in ion transport in *C. rodentium*-infected SW mice, including subtle decreases in expression of some apical transporters and compensatory increases in basolateral water channel *Aqp4 *expression. **(b) **Profound changes in infected FVB mice consisting of mild to marked downregulation of the majority of apical transporters involved in intestinal Na^+ ^and Cl^- ^absorption and bicarbonate secretion, along with upregulation of basolateral transporters providing the driving force for chloride secretion. In addition, constitutively higher levels of *Pept2 *and *Adora2b *expression in FVB mice (indicated by asterisks) can contribute to alterations in cytosolic pH and cAMP during infection, thereby further affecting ion exchange. The cumulative effect may ultimately result in severe diarrhea and lead to death in these susceptible animals. Vesicular trafficking of some proteins (A2B receptor, aquaporins, NHE3, ATPases) and paracellular transport are not addressed here. Colors indicate fold change in gene expression identified by microarray or qRT-PCR: light green ≥2-fold decrease; dark green ≥8-fold decrease; pink ≥2-fold increase; red ≥8-fold increase.

Genes differentially expressed between FVB and SW mice, including the most significant candidate susceptibility genes, were highly enriched for transport activity (Figure 3c; Additional data files 10 and 13). These results demonstrated a larger fraction of transport genes than immune-related genes in determining susceptibility to *C. rodentium *infection and were confirmed using different computational algorithms (Figures 3c; Additional data file 16). In addition, serum chemistry analysis showed significant hypochloremia and hyponatremia in infected FVB mice (Figure 6), consistent with marked electrolyte losses in animals suffering from severe diarrhea [17,20,21]. We previously suggested that mortality in FVB mice infected with *C. rodentium *could be attributed to hypovolemia induced by severe diarrhea [13]. Results from this study suggest that intestinal ion disturbances rather than immune-related processes are responsible for the dramatic phenotype in *C. rodentium*-infected FVB mice. Here, we discuss potential candidate genes for susceptibility identified by microarray analysis and how they could contribute to mortality in FVB mice infected with *C. rodentium*. Our working hypothesis for the overall mechanism of susceptibility is presented in Figure 7.

### Candidates for susceptibility: genes involved in ion transport and its regulation

The main function of the adult colon is to absorb Na^+^, Cl^-^, K^+^, short-chain fatty acids (SCFAs) and fluid and to secrete HCO_3_^- ^and mucus (reviewed in [15,16]). The osmotic gradient created by active salt transport using ion transporters and channels is the driving force for passive water movement in/out of the lumen. Water transport can also occur actively through specific water channels, aquaporins. The absorptive functions of the colon are usually dominant, whereas during diarrhea transport favors more electrolyte secretion and less absorption, resulting in loss of fluid into intestinal lumen [15,16]. Secretion of fluid and mucus into the lumen is an important mucosal defense mechanism that serves to dilute and wash away injurious substances from the epithelial surface [22]. However, these mechanisms also can lead to hypovolemia, circulatory collapse and multiple organ failure when profound fluid losses result in marked decrease in plasma volume [3].

In FVB mice, transcriptional changes in transporters and signaling or regulatory genes were more dramatic than in SW mice during *C. rodentium *infection, particularly at 9 dpi (Figure 7).

#### Chloride absorption

The most remarkable difference between infected FVB mice and SW mice was in *Slc26a3*, which encodes the down-regulated in adenoma (Dra) protein. Dra mediates apical sodium-independent reabsorption of chloride into epithelial cells and excretion of bicarbonate into the lumen [23]. Mutation in Dra is associated with congenital chloride diarrhea, a recessive inherited intestinal disorder characterized by watery diarrhea and severe dehydration, high levels of fecal chloride, electrolyte disturbances, including hypochloremia and hyponatremia, leading to metabolic alkalosis, hypokalemia, and death if untreated [15,23]. The *Dra*^-/- ^mouse is the only mouse model for intestinal transporter deficiency that develops substantial diarrhea and serum electrolyte imbalances [17], whereas *NHE2*^-/-^, *NHE3*^-/-^, *cHKA*^-/-^, *Aqp4*^-/- ^and *Aqp8*^-/- ^mice have subtle, if any, changes in stool water content and normal serum levels of chloride and sodium [24-29]. In addition, a recent report described inhibition of intestinal apical chloride absorption mediated by DRA in Caco-2 cells infected with EPEC [30]. Dramatic downregulation of *Dra *mRNA (by 1,100-fold at 9 dpi) and lack of Dra expression in the distal colon of infected FVB mice strongly implicate *Slc26a3 *as a susceptibility factor in fatal diarrhea induced by AE pathogens.

#### Chloride secretion

There are conflicting data regarding regulation of *Slc26a3 *by cystic fibrosis transmembrane conductance regulator (CFTR), the major chloride channel [31-34]. In this study, expression of *Cftr *was only slightly decreased in FVB mice at 9 dpi and was not associated with changes in *Slc26a3 *mRNA. Similar observations were made by Umar *et al*. [35], who reported subtle changes in *Cftr *expression in whole distal colon from SW mice infected with *C. rodentium*. Although isolated crypts exhibited a greater increase in the levels of *Cftr *mRNA and protein, this did not correlate with large increases in transmucosal cAMP-dependent Cl^- ^current [35], suggesting that other factors play an important role in regulating chloride transport during *C. rodentium *infection. For example, chloride secretion via CFTR and other chloride channels can be activated by cAMP, cGMP, and calcium [5,36]. *Camk2b *and *Prkg2*, which would be expected to be downstream of these signals, were increased in infected FVB but not SW mice. This is consistent with a post-transcriptional mechanism of CFTR involvement in *C. rodentium*-induced diarrhea. Notably, the same signaling pathways are also implicated in inhibition of sodium/proton exchange and electrogenic Na^+ ^absorption in colon [16,37].

#### Sodium absorption

Expression of *ENaC*, *NHE2 *and *NHE3*, which are the main sodium channels involved in apical electrogenic and electroneutral sodium absorption, pH maintenance and fluid balance in intestine [16,37], was decreased by infection in FVB mice, and to a lesser extent in SW mice. Downregulation of the calcineurin B homologous protein (CHP), which is required for Na^+^/H^+ ^exchange activity [38], further indicates that intestinal transport is affected at the transcriptional and post-transcriptional levels in infected FVB mice. Coupling of DRA, CFTR, and NHE3 activity is mediated by PDZ-binding scaffold proteins (reviewed in [39]). One such protein, EBP50, encoded by *Slc9a3r1 *(*NHERF1*), is required for cAMP-mediated inhibition of NHE3 activity and stimulation of CFTR activity. The interaction of NHERF1 with the type III secretion system effector Map was recently implicated in the pathogenesis of EPEC and *C. rodentium *[40]. The decrease in expression of EBP50 in infected FVB mice may represent an unsuccessful attempt to control diarrhea. It is notable that decreased expression of individual sodium transport genes was mild (2- to 13-fold), but collectively the failure to reabsorb sodium contributes to mortality.

#### Potassium transport

The driving force for apical Cl^- ^secretion is dependent on basolateral potassium recycling through Na^+^,K^+^-ATPase and K^+ ^channels [36]. Infected FVB mice demonstrated marked increases in expression of *Atp1b2*, which encodes Na^+^,K^+^-ATPase beta-2 isoform at both 4 and 9 dpi, and to a lesser extent increased expression of potassium channels *KCNN4 *and *NKCC1 *at 9 dpi. Furthermore, Na^+^,K^+^-ATPase activity was recently shown to be indirectly stimulated by *SNX6 *[41]. This gene was not only consistently overexpressed in FVB mice at all times compared to SW mice, but also was upregulated in FVB mice at 4 and 9 dpi. These changes could potentiate electrogenic Cl^- ^secretion and, hence, fluid loss. While basolateral potassium transporters were mainly upregulated in infected FVB mice, the luminal ouabain-sensitive H^+^,K^+^-ATPase (cHKA) encoded by *Atp12a *was downregulated in FVB mice but not in SW mice at 9 dpi. This ATPase was reported to regulate Dra activity in the colons of NHE3-deficient mice [42]. Furthermore, compensatory increases in intestinal *cHKA *expression have been observed in a number of ion transporter gene knockout mouse models [17,27], indicating its role in maintaining intestinal ion homeostasis. Thus, impaired transcriptional activity of potassium transporters can contribute to the pathogenesis of diarrhea in *C. rodentium*-susceptible animals.

#### Bicarbonate metabolism and pH regulation

The activity of many intestinal transporters is regulated by intracellular pH. Rapid diffusion and equilibration of protons entering enterocytes at the apical membrane is dependent on carbonic anhydrases, enzymes that catalyze the reversible hydration/dehydration of CO_2 _and water [43]. Carbonic anhydrases, especially cytosolic CA I and membrane-associated CA IV, are known to play a role in ion and water transport in the small intestine and distal colon [44-47]. Because inhibition of carbonic anhydrases is associated with marked decreases in sodium, chloride and water absorption as well as bicarbonate secretion [47-49], profound downregulation of *CA I *and *CA IV *in infected FVB mice at 9 dpi suggest their critical role in *C. rodentium*-induced mortality.

Additional proteins able to affect intracellular pH include oligopeptide-proton symporters *Pept1 *and *Pept2*, whose expression was differentially expressed between FVB and SW mice. Although H^+^/dipeptide transport is coupled with Na^+^/H^+ ^exchanger and carbonic anhydrase activity in Caco-2 cells and mouse enterocytes isolated from small intestine [43,50], the function of these transporters in distal colon is not clear [51,52].

#### SCFA transport

Both cytosolic and luminal pH is regulated by butyrate and other SCFAs produced by enteric bacterial fermentation of undigested carbohydrates and dietary fiber. SCFAs, following ileal or colonic absorption by nonionic diffusion or via a SCFA/HCO_3_^-^(OH) exchange mechanism, maintain mucosal integrity and stimulate water and electrolyte absorption by acidification of colonocytes and activation of apical Na^+^/H^+ ^and Cl^-^/HCO_3_^- ^exchangers [45,53,54]. Therefore, decreased butyrate/SCFAs availability due to downregulation of the Na^+^-dependent SCFA transporter *Slc5a8 *(*Ait*) in FVB mice at 9 dpi might affect mucosal permeability, disturb acid-base homeostasis, and inhibit ion absorption in the colon, thereby contributing to *C. rodentium*-mediated diarrhea in susceptible FVB mice. This is similar to the decreased expression of the epithelial SCFA transporter MCT-1, and the subsequent decrease in butyrate uptake reported in Caco-2 cells infected with EPEC [53]. This inhibitory effect is dependent on a functional type III secretion system (*escN*, *espA*, *espB*, or *espD *genes), but did not require known effector proteins encoded by *espF*, *espG*, *espH*, or *map *[53]. These results suggest that intimate attachment and perhaps a yet unidentified bacterial effector protein(s) are necessary for decreased butyrate uptake in host cells.

#### Water transport

As mentioned previously, water passes through cells either passively, following the osmotic gradient created by chloride and sodium transport, or is transported by means of specific water channels, aquaporins [55]. A number of aquaporins were differentially expressed in infected FVB mice compared with SW mice. Apical channel *Aqp8 *was profoundly downregulated in FVB mice at 9 dpi, which could interfere with fluid transport from the intestinal lumen in susceptible animals. In resistant SW mice, fluid loss caused by decreased ion reabsorption is compensated for by upregulation of the basolateral channel *Aqp4*, which counteracts movement of water into the lumen. Although a slight increase in *Aqp4 *expression was observed in FVB mice at 4 dpi, the transcript levels returned to baseline at 9 dpi. These results are consistent with a recent report implicating mislocalization of aquaporins 2 and 3 in *C. rodentium *diarrhea [56]. Thus, the function of water channels in *C. rodentium*-infected FVB mice deserves further investigation.

#### Transcriptional regulation

Among the transcription factors differentially expressed between susceptible and resistant mice, *Fosb *was the most affected early in infection. The upregulation of *Fosb *in infected FVB mice at 4 dpi was more appreciable than the subtle increase observed in SW mice at that time point. This trend continued at 9 dpi as well. FosB is an 'immediate-early' nuclear protein from the Fos family of transcription factors. Dimerization of Fos and Jun proteins causes binding to activator protein 1 (AP-1) promoter sites. AP-1 complexes affect expression of many genes, including ion channels, receptors, cytoskeletal proteins and signaling molecules [57,58]. Within the set of genes with a strain effect, 67% were predicted to have AP-1 binding sites. This included many potential candidates for susceptibility, such as *Aqp4 *and *Aqp8*, *cHKA*, *CA I*, *Slc26a3*, *Slc5a8*, *Slc9a2*, *NHERF1*, and *Fosb *itself (data not shown). Early upregulation of *Fosb *in infected FVB mice may indicate that FosB contributes to fatal diarrhea independent of, and prior to, inflammation-mediated effects. Further studies are needed to determine the signals that upregulate *Fosb *expression in susceptible mice, FosB partners in DNA binding and FosB target genes.

#### Inflammatory effectors

Inflammatory mediators are also implicated in the pathogenesis of diarrhea. A good example is adenosine, a secretagogue released by polymorphonuclear cells, eosinophils and mast cells in inflammatory conditions [59-61]. Breakdown of ATP released from injured cells during infection is an additional possible source of adenosine, as has been proposed for pathogens with type III secretion systems such as EPEC, *E. coli *O157:H7, and *Salmonella enterica *[62]. Adenosine signaling is mediated by G-protein-coupled receptors, of which A2BAR encoded by *Adora2b *is the predominant adenosine receptor in intestinal epithelial cells [61]. Downstream signaling by activated A2BAR results in cAMP- and arachidonic acid-dependent activation of potassium channels and intestinal Cl^- ^secretion [59,61,63,64]. The consistently higher *Adora2b *expression in FVB mice may predispose susceptible animals to this diarrhea-inducing pathway and contribute to the susceptibility of FVB mice to *C. rodentium *infection.

#### Epithelial differentiation

Notably, a number of genes profoundly downregulated in susceptible FVB mice, such as *Slc26a3 *(*Dra*), *Aqp8*, *CAIV*, and *Slc5a8 *(*Ait*), are also implicated in the development of colonic tumors [65-68]. Thus, relative loss of differentiated epithelium due to erosions and ulcerations and/or expansion of less well differentiated proliferating cells in the crypt compartment [13] could contribute to altered transcriptional activity in infected FVB mice. However, expression of other markers of differentiation, such as *sucrase-isomaltase*, *alkaline phosphatase*, *villin*, *intestinal trefoil factor 3*, and *Kruppel-like factor 4 *[69,70], was unchanged or not altered to the same extent in infected FVB mice, indicating that erosions or hyperplasia alone can not fully account for the dramatic loss of expression of *Dra*, *Aqp8*, *CAIV*, and *Ait *in susceptible FVB mice.

#### Developmental regulation

Expression of many genes identified in our global analysis change during postnatal development. This includes sodium exchangers, aquaporins and carbonic anhydrases with higher levels of expression and PEPT proteins with lower expression in adults animals compared with suckling or weanling animals [52,71-75]. This suggests that mortality in *C. rodentium*-infected adult FVB mice and young mice of all strains and stocks may result from a common pathogenic mechanism, such as inadequate apical ion, proton and water transport in the distal colon leading to dehydration and hypovolemic shock. Therefore, genes identified by microarray analysis deserve further study and may account for susceptibility to fatal infectious diarrhea in young mice and other mammals.

#### Applications to other models of diarrhea

Ion imbalances are implicated in other non-infectious animal models of diarrhea. Thus, mice with defects in cytoskeletal intermediate filaments, like keratin 8-deficient mice (in an FVB/N background), demonstrate ion transport impairment before the onset of colonic hyperproliferation and inflammation. Interestingly, those animals develop diarrhea despite normal tight junction permeability [76], raising the possibility that tight junction abnormalities contribute to, but do not directly cause, mortality in *C. rodentium*-infected FVB mice. In addition, significant impairment of sodium and chloride absorption and bicarbonate secretion is found in colitis-prone IL-2^-/- ^mice [77]. Chemical induction of colitis by treatment with dextran sulfate sodium results in substantial downregulation of carbonic anhydrases *CA I *and *CA IV *and aquaporins *Aqp4 *and *Aqp8 *[78-80]. These results indicate that infectious diarrhea and noninfectious inflammation-associated diarrhea may have common mechanisms of pathogenesis and further justify the use of *C. rodentium*-infected FVB mice for studying fluid and electrolyte imbalance.

## Conclusion

We present the first gene expression profiling of *C. rodentium *infection *in vivo*. The genomic analysis of the host response to infection generated novel testable hypotheses regarding this enteric murine pathogen's ability to cause disease and mortality in FVB mice. Marked impairment in intestinal ion homeostasis was predicted by microarray analysis and confirmed by qRT-PCR, immunohistochemistry and serum electrolyte measurements. The fact that the majority of genetically manipulated mice with a single deficiency in ion transporters develop only mild, if any, diarrhea and no appreciable serum electrolyte disturbances indicates the existence of compensatory mechanisms. It is likely that in the disease state (for example, diarrhea induced by *C. rodentium*) many genes involved in intestinal ion transport, signaling and regulation act together. In that regard, orchestrated alterations, such as downregulation of the main apical colonic transporters, upregulation of basolateral ion channels and other changes in regulatory signals observed in susceptible FVB mice upon *C. rodentium *infection, may provide a basic mechanism for the development of severe diarrhea and fatal dehydration in susceptible strains compared with resistant strains of mice. Our study identified potential candidate genes for susceptibility that can be used to develop new strategies for preventing and treating intestinal inflammation and fatal diarrhea.

## Materials and methods

### Media, bacterial strains, and growth conditions

Lennox L (LB) broth and LB agar (Difco Laboratories, Detroit, MI, USA) were used for routine cultivation of bacteria. MacConkey lactose agar (Difco Laboratories) supplemented with 40 μg/ml of kanamycin was used for quantitative microbiology of fecal samples. The kanamycin-resistant *C. rodentium *strain DBS120 (pCRP1::Tn*5*, Kan^r^) [13] was used for infections.

### Animal infections

Inbred FVB/NTac and outbred Swiss Webster Tac:SW females with 40 and 36 female two-week-old pups, respectively, were purchased from Taconic Laboratories (Germantown, NY, USA). Because animals came from different barrier units, mixing of bedding from weaning until the time of inoculation (12 weeks of age) was performed twice a week to obtain comparable microbial status and minimize commensal microbiota biases. Animals were housed in microisolator cages in a specific pathogen-free facility approved by the Association for Assessment and Accreditation of Laboratory Animal Care and maintained on pelleted rodent chow (LabDiet, Purina Mills, Inc., Richmond, IN, USA) and water *ad libitum*. At 12 weeks of age, infectious colitis was induced by intragastric inoculation with 1.9 × 10^9 ^CFU of DBS120 as described previously [13]. A total of 37 FVB and 36 SW mice were used in the inoculation study as described in Additional data file 1. Animals were weighed and monitored for fecal bacterial shedding prior to inoculation and at 3, 6, and 8 dpi. The lower limit of detection for quantitative microbiology was 1 CFU/mg of feces. Animals were euthanized at 4 and 9 dpi. At necropsy, the colon of each mouse was collected aseptically, feces were removed from the lumen, and the distal colon was transected in-half longitudinally. Half of the distal colon was snap frozen in liquid nitrogen and stored at -80°C until RNA was extracted. The rest of the tissue was fixed in 10% neutral-buffered formalin for 24-48 hours, processed routinely, paraffin embedded, sectioned at 5 μm, and stained with hematoxylin and eosin. Sections were scored for lesions on a scale of 0 to 4 (none, minimal, mild, moderate, and severe) by a veterinary pathologist (PRN) blinded to experimental groups. All experiments were approved by the MIT Animal Care and Use Committee.

### RNA extraction

Total RNA was extracted from frozen distal colon using Trizol reagent according to the recommendations of the manufacturer (Invitrogen, Carlsbad, CA, USA). RNA was treated with DNase I and purified using an RNeasy Clean-up kit as recommended by the manufacturer (Qiagen, Valencia, CA, USA). The total RNA concentration and 260/280 ratio was evaluated spectrophotometrically. Only samples with a 260/280 ratio between 1.8 and 2.1 were further processed. RNA samples were evaluated using an Agilent 2100 Bioanalyzer (Agilent, Palo Alto, CA, USA) and consistently demonstrated high-quality RNA with distinct 28S and 18S peaks and no evidence of degradation.

### Array design and hybridization

Global gene expression analysis was performed on the distal colon with two to three mice per group. Because no difference for any parameters was observed in uninfected mice at 4 or 9 dpi, the animals were pooled into an uninoculated control group for each line of mouse. The selection of representative samples for microarray analysis was based on known infection status and colonic lesions. The final number of biological replicates for each condition was n = 5 for uninoculated FVB mice ('Fp' group), n = 4 for uninoculated SW mice ('Sp' group), and n = 3 for infected animals from each line at each time point ('Fi4', 'Si4', 'Fi9', and 'Si9', respectively; Additional data file 1). One-cycle target labeling of isolated RNA, hybridization, washing/staining and scanning was carried out in the Whitehead Institute Center for Microarray Technology (Cambridge, MA, USA) as detailed at [81]. First- and second-strand cDNA syntheses were performed using SuperScript double-stranded cDNA synthesis kit (Invitrogen). Second strand DNA synthesis, clean-up of the double-stranded cDNA, and synthesis and clean-up of biotin-labeled cRNA were completed according to Affymetrix protocols (Santa Clara, CA, USA). cRNA (20 μg) was fragmented and hybridized as recommended by Affymetrix to the GeneChip^® ^Murine Genome 430 2.0 Arrays containing 45,037 probe sets that correspond to over 34,000 well characterized mouse genes. Each sample was hybridized to one array, using a total of 21 chips. Arrays were scanned using a GeneChip scanner 3000, enabling for high-resolution scanning as recommended (Affymetrix). The expression output for all samples met quality control requirements (data not shown). Presence call for all arrays ranged from 58-66%. Microarray results were tightly correlated between biological replicates within and between the animal groups (Additional data file 21).

### Identifying differentially expressed genes

Cell intensity files (*.cel) containing hybridization signals were generated with GeneChip Operating Software (GCOS 1.2). Normalization and processing were carried out using DNA-Chip Analyzer (dChip) software [82] implementing model-based expression index analysis using an outlier detection algorithm to eliminate potential cross-hybridizing probes [83]. Normalization using an invariant set of genes, in which all arrays are normalized to a common baseline array with median intensity, was followed by background correction and log2 transformation. The perfect match-only model was applied in order to reduce noise. A mean value was calculated from signal log2 ratios for each gene and group. Gene expression was considered to be significant when it was changed by more than two-fold (an average log2 ratio above 1.0 or below -1.0). Genes were clustered by tightness with centroid-linkage method using 1 - r (where r is the Pearson correlation coefficient) as the distance measure while redundant probes were masked. Results were visualized using heat maps showing color-coded expression levels (red = high expression, black = medium expression, and green = low expression) and vertically drawn gene trees. Functional enrichment analysis was performed on non-redundant genes with known functions using two methods; within dChip software using a hypergeometric test with *p *< 0.05 and at least four functionally annotated genes if not otherwise indicated. In addition, the web-based Fast Assignment and Transference of Information using Gene Ontology (FatiGO) Plus tool [84,85] was used for calculating the prevalence of GO functional groups. The results of inclusive analysis at the fifth level of depth and more than 5% of GO categories enrichment are presented. Temporal changes in response to infection were addressed using a 'delta eta' analysis to identify significant differential transcript modulation in response to treatment where for each transcript [log2 (FVB infected/FVB uninoculated) - log2 (SW infected/SW uninoculated)] where FC >1.5. For each gene, delta eta values for 4 and 9 dpi were calculated as log2(Si4 versus Sp) - log2(Fi4 versus Fp) and log2(Si9 versus Sp) - log2(Fi9 versus Fp), respectively. The results were processed in Spotfire and subjected to enrichment GO analysis by dChip. All Affymetrix and GO annotations were based on February 2007 data files.

Results obtained by dChip data processing were compared to other analytical methods: the raw CEL files were processed using the Robust Multichip Average algorithm [86] and differentially expressed genes were identified using linear modeling with a moderated *t*-statistic for each gene (Limma package) [87], available as part of BioConductor software. Temporal infection-induced trends between the groups were determined by a linear model with false discovery rate <0.05 and 2-fold cut-off threshold. To discriminate gene behavior, random forest analysis to predict four classes (strain × infection status) was applied with *p *< 0.05. Enrichment analysis was performed within dChip with *p *< 0.0005, and the prevalence of functional groups was determined with FatiGO as indicated above.

Raw data and normalized microarray expression data have been deposited at the Gene Expression Omnibus (GEO) [88] under the accession number GSE8025.

### TaqMan quantitative RT-PCR

Total RNA (5 μg) was used to generate cDNA with SuperScriptII RT (Invitrogen) as recommended by the manufacturer. cDNA (100 ng) was amplified in a 25 μl reaction volume with Applied Biosystems (Branchburg, NJ, USA) predesigned primers and probes (TaqMan Gene Expression Assays; Additional data file 3) in an ABI Prism Sequence Detection System 7700 (Applied Biosystems) using standard TaqMan protocols. Transcript levels were normalized to the endogenous control glyceraldehyde-3-phosphate dehydrogenase (GAPDH), and expressed as fold change compared with averaged uninoculated control FVB mice, which were set at 1, using the Comparative Ct method [89]. The resultant ratios were matched with corresponding comparisons from microarray analysis and subjected to Pearson correlation analysis. The number of samples used for TaqMan were n = 10 in uninoculated control groups and n = 6-8 samples for infected groups in each line of mice (Additional data file 1). Each reaction was carried out in duplicate.

### Immunohistochemical analysis

The expression of Dra and CA IV in formalin fixed paraffin-embedded tissues was detected using rabbit anti-Slc26a3 (kind gift from Dr Schweinfest [17], diluted 1:100) and goat anti-CA IV (AF2414, R&D Systems, Minneapolis, MN, USA; diluted 1:800) polyclonal antibodies. After heat induced epitope retrieval (pH 6), primary Dra antibodies were detected with biotinylated goat anti-rabbit IgG (E0432, Dako, Carpinteria, CA, USA) and normal rabbit IgG (x0936, Dako) was used as a negative control. CA IV antibodies were detected with biotinylated rabbit anti-goat IgG (b7014, Sigma, St. Louis, MO, USA) and goat HRP-polymer kit (ghp516g, Biocare Medical, Concord, CA, USA), whereas normal goat IgG (#005-000-003, Jackson Immuno, West Grove, PA, USA) was used as a negative control. Sections were stained using diaminobenzidine as a substrate and counterstained with hematoxylin.

### Measuring serum electrolyte levels

Electrolytes in serum were assayed by IDEXX Preclinical Research Services (IDEXX Laboratories, Inc., North Grafton, MA, USA) using electrolyte Panel 957, including bicarbonate, chloride, potassium, sodium, Na^+^/K^+ ^ratio and anion gap, with 200 μl samples of serum.

### Statistics

Data are presented as mean values ± the standard error of mean or median values (for nonparametric data). Statistical analyses were performed using GraphPad PRISM version 4.0 (GraphPad Software, Inc., San Diego, CA, USA) or JMP 5.0.1 software (SAS Institute Inc., Cary, NC, USA). Statistical differences were determined by using nonparametric Kruskal-Wallis test followed by Dunn's Multiple Comparison test or with one-way ANOVA followed by Student's *t*-test or Tukey's Multiple Comparison Test. Whenever Bartlett's test showed unequal variances, analysis of gene expression was performed on transformed data. A *p*-value < 0.05 was regarded as statistically significant.

## Abbreviations

A/E, attaching and effacing; AP, activator protein; AQP, aquaporin; CA, carbonic anhydrase; CFTR, cystic fibrosis transmembrane conductance regulator; dChip, DNA-Chip Analyzer; dpi, days post inoculation; Dra, down-regulated in adenoma; EHEC, enterohaemorrhagic *E. coli*; EPEC, enteropathogenic *E. coli*; FatiGO, Fast Assignment and Transference of Information using Gene Ontology; FVB, FVB/N mice; GO, Gene Ontology; NHE, Na/H exchanger; PC, principle component; PCA, principal component analysis; qRT-PCR, quantitative real-time PCR; SCFAs, short-chain fatty acids; SW, Swiss Webster.

## Authors' contributions

DB conceived of the studies, designed them, performed the statistical analysis, and drafted the manuscript. RCF participated in the design of the study and bioinformatic analysis. EBG carried out the genomic studies. PRN scored the histopathological slides. VJC participated in bioinformatic analysis. JGF conceived of the studies and participated in manuscript drafting. DBS conceived of the studies, participated in their design and coordination, and drafted the manuscript. All authors read and approved the final manuscript.

## Additional data files

The following additional data are available. Additional data file 1 is a table listing the number of animals used for experiments. Additional data file 2 is a table listing all differentially expressed genes. Additional data file 3 is a table displaying validation of microarray results by quantitative RT-PCR (TaqMan) on selected genes. Additional data file 4 is a figure showing side-by-side comparison of gene expression analyzed by microarray and qRT-PCR. Additional data file 5 is a table listing genes with host effect. Additional data file 6 is a table listing PCA parameters. Additional data file 7 is a figure showing hierarchical clustering of genes with host effect. Additional data file 8 is a table listing the most significant variably expressed genes with host effect. Additional data file 9 is a figure showing hierarchical clustering of the most differentially expressed genes with host effect. Additional data file 10 is a table summarizing enrichment by GO categories of the most differentially expressed genes with host effect. Additional data file 11 is a table listing the variably expressed genes as a function of time (delta eta analysis). Additional data file 12 is a figure showing hierarchical clustering of the most differentially expressed genes from delta eta analysis. Additional data file 13 is a table summarizing enrichment by GO categories of the most differentially expressed genes from delta eta analysis. Additional data file 14 is a table listing the genes with host effect analyzed by BioConductor. Additional data file 15 is a table listing the genes with host × infection effect analyzed by BioConductor. Additional data file 16 is a figure showing FatiGO analysis on genes identified by BioConductor analysis. Additional data file 17 is a table summarizing enrichment by GO categories of genes detected by BioConductor analysis. Additional data file 18 is a figure showing genes with most predictive power obtained from BioConductor analysis. Additional data file 19 is a figure presenting hierarchical clustering of genes discussed in text as potentially contributing to development of intestinal ion disturbances and diarrhea. Additional data file 20 is a table listing enriched GO categories in the set of genes with host effect and by groups indicated in Figure 3a. Additional data file 21 is a figure showing correlation of raw intensities between biological replicates validating microarray results.

## Supplementary Material

Additional data file 1 Number of animals used for experiments. Click here for file

Additional data file 2 All differentially expressed genes. Click here for file

Additional data file 3 Validation of microarray results by quantitative RT-PCR (TaqMan) on selected genes. Click here for file

Additional data file 4 Side-by-side comparison of gene expression analyzed by microarray and qRT-PCR. Click here for file

Additional data file 5 Genes with host effect. Click here for file

Additional data file 6 PCA parameters. Click here for file

Additional data file 7 Hierarchical clustering of genes with host effect. Click here for file

Additional data file 8 The most significant variably expressed genes with host effect. Click here for file

Additional data file 9 Hierarchical clustering of the most differentially expressed genes with host effect. Click here for file

Additional data file 10 Enrichment by GO categories of the most differentially expressed genes with host effect. Click here for file

Additional data file 11 Variably expressed genes as a function of time (delta eta analysis). Click here for file

Additional data file 12 Hierarchical clustering of the most differentially expressed genes from delta eta analysis. Click here for file

Additional data file 13 Enrichment by GO categories of the most differentially expressed genes from delta eta analysis. Click here for file

Additional data file 14 Genes with host effect analyzed by BioConductor. Click here for file

Additional data file 15 Genes with host × infection effect analyzed by BioConductor. Click here for file

Additional data file 16 FatiGO analysis on genes identified by BioConductor analysis. Click here for file

Additional data file 17 Enrichment by GO categories of genes detected by BioConductor analysis. Click here for file

Additional data file 18 Genes with most predictive power obtained from BioConductor analysis. Click here for file

Additional data file 19 Hierarchical clustering of genes discussed in text as potentially contributing to development of intestinal ion disturbances and diarrhea. Click here for file

Additional data file 20 Enriched GO categories in the set of genes with host effect and by groups indicated in Figure 3a. Click here for file

Additional data file 21 Correlation of raw intensities between biological replicates validating microarray results. Click here for file
