# Supplementary material for: Diarrhea as a cause of mortality in a mouse model of infectious colitis
Source: Genome Biol. 2008 Aug 4;9(8):R122. doi: 10.1186/gb-2008-9-8-r122 (PMC2575512; doi:10.1186/gb-2008-9-8-r122)
Supplement: Additional data file 1 — Number of animals used for experiments. [file gb-2008-9-8-r122-S1.doc]

| **Additional data file 1.** Number of animals used for microarray analysis, quantitative RT-PCR (TaqMan) and experiment | | | | | | |
| --- | --- | --- | --- | --- | --- | --- |
| Array sample name | Days post inoculation | Group | Group label | Total number of arrays in a group | Total number of samples for TaqMan | Total number of mice in a group |
| S4_1  S4_2  S9_1  S9_2 | 4  4  9  9 | SW pooled controls | Sp | 4 (2 + 2) | 10 (5 + 5) | 16 (8 + 8) |
| F4_1  F4_2  F9_1  F9_2  F9_3 | 4  4  9  9  9 | FVB pooled controls | Fp | 5 (2 + 3) | 10 (5 + 5) | 20 (10 + 10) |
| Si4_1  Si4_2  Si4_3 | 4  4  4 | SW 4 dpi | Si4 | 3 | 6 | 10 |
| Fi4_1  Fi4_2  Fi4_3 | 4  4  4 | FVB 4 dpi | Fi4 | 3 | 8 | 10 |
| Si9_1  Si9_2  Si9_3 | 9  9  9 | SW 9 dpi | Si9 | 3 | 6 | 10 |
| Fi9_1  Fi9_2  Fi9_3 | 9  9  9 | FVB 9 dpi | Fi9 | 3 | 7 | 7 |
