# Supplementary material for: Diarrhea as a cause of mortality in a mouse model of infectious colitis
Source: Genome Biol. 2008 Aug 4;9(8):R122. doi: 10.1186/gb-2008-9-8-r122 (PMC2575512; doi:10.1186/gb-2008-9-8-r122)
Supplement: Additional data file 3 — Validation of microarray results by quantitative RT-PCR (TaqMan) on selected genes. [file gb-2008-9-8-r122-S3.doc]

| **Additional data file 3.** Validation of microarray results by quantitative RT-PCR (TaqMan) on selected genes | | | | | |
| --- | --- | --- | --- | --- | --- |
| Gene | Gene Symbols | ABI ID for predesigned assay-on-demand | Pearson correlation | P value | R^2^ of linear fit |
| adenosine A2b receptor | Adora2b | [Mm00839292_m1](http://myscience.appliedbiosystems.com/servlet/com.celera.web.cdsentry.servlets.GetCdsEntryListServlet?cmd=cmdGetGeneExDetailReport&assayAcc=Mm01315577_s1) | 0.91 | 0.0007 | 0.83 |
| angiogenin, ribonuclease A family, member 4 | Ang4, Rnase5d | [Mm01315577_s1](http://myscience.appliedbiosystems.com/servlet/com.celera.web.cdsentry.servlets.GetCdsEntryListServlet?cmd=cmdGetGeneExDetailReport&assayAcc=Mm01315577_s1) | 0.97 | <0.0001 | 0.95 |
| aquaporin 8 | Aqp8 | Mm00431846_m1 | 0.97 | <0.0001 | 0.95 |
| arachidonate 12-lipoxygenase | Alox12 | Mm00545833_m1 | 0.98 | <0.0001 | 0.96 |
| arginase type I, liver | Arg1 | [Mm00475988_m1](http://myscience.appliedbiosystems.com/ge/servlet/com.celera.web.cdsentry.servlets.GetCdsEntryListServlet?cmd=cmdGeReport&assayType=geneExpression&assayAcc=Mm00475988_m1) | 0.93 | 0.0002 | 0.87 |
| arginase type II | Arg2 | Mm00477592_m1 | 0.88 | 0.0019 | 0.77 |
| ATPase, Na+/K+ transporting, beta 2 polypeptide | Atp1b2 | Mm00442612_m1 | 0.89 | 0.0014 | 0.79 |
| beta galactoside alpha 2,6 sialyltransferase 1 | Siat1, ST6GaI1 | Mm00486119_m1 | 0.86 | 0.0028 | 0.74 |
| bone morphogenetic protein 5 | Bmp5, se | Mm00432091_m1 | 0.94 | 0.0001 | 0.89 |
| carbonic anhydrase 1 | Car1, CA I | Mm00486717_m1 | 0.99 | <0.0001 | 0.99 |
| carbonic anhydrase 4 | Car4, CA IV | Mm00483021_m1 | 1.00 | <0.0001 | 0.99 |
| CD14 antigen | Cd14 | Mm00438094_g1 | 0.91 | 0.0007 | 0.83 |
| CD34 antigen | Cd34 | Mm00519283_m1 | 0.90 | 0.0009 | 0.81 |
| complement receptor related protein | Crry | Mm00785297_s1 | -0.38 | 0.3123 | 0.14 |
| cystic fibrosis transmembrane conductance regulator homolog | CFTR, Abcc7 | Mm00445197_m1 |  |  |  |
| extracellular proteinase inhibitor | Expi, WDNM1 | Mm00433159_m1 | 0.98 | <0.0001 | 0.97 |
| FBJ osteosarcoma oncogene B | FosB | Mm00500401_m1 | 0.89 | 0.0014 | 0.79 |
| glyceraldehyde-3-phosphate dehydrogenase | GAPDH | Mm99999915_g1 |  |  |  |
| histocompatibility 2, class II antigen E beta | H2-Eb1, IIa-4 | [Mm00439221_m1](http://myscience.appliedbiosystems.com/servlet/com.celera.web.cdsentry.servlets.GetCdsEntryListServlet?cmd=cmdGetGeneExDetailReport&assayAcc=Mm00439221_m1) | 0.86 | 0.0027 | 0.75 |
| immunoglobulin lambda chain, variable 1 | Igl-V1 | Mm01627739_g1 | 0.81 | 0.0086 | 0.65 |
| Intelectin | Itln | Mm01616618_g1 | 0.67 | 0.0468 | 0.45 |
| Interferon activated gene 202B | Ifi202b | [Mm00839397_m1](http://myscience.appliedbiosystems.com/servlet/com.celera.web.cdsentry.servlets.GetCdsEntryListServlet?cmd=cmdGetGeneExDetailReport&assayAcc=Mm00839397_m1) | 0.97 | <0.0001 | 0.93 |
| Interferon activated gene 203 | Ifi203 | [Mm01238610_m1](http://myscience.appliedbiosystems.com/servlet/com.celera.web.cdsentry.servlets.GetCdsEntryListServlet?cmd=cmdGetGeneExDetailReport&assayAcc=Mm00839397_m1) | 1.00 | <0.0001 | 0.99 |
| Interferon activated gene 205 | Ifi205 | [Mm01315309_m1](http://myscience.appliedbiosystems.com/servlet/com.celera.web.cdsentry.servlets.GetCdsEntryListServlet?cmd=cmdGetGeneExDetailReport&assayAcc=Mm01315309_m1) | 0.75 | 0.0189 | 0.57 |
| lectin, galactose binding, soluble 6 | Lgals6 | Mm02524460_s1 | 0.92 | 0.0005 | 0.85 |
| mucin 2 | muc2 | Mm00458304_g1 | 0.98 | <0.0001 | 0.96 |
| myelocytomatosis oncogene | Myc | Mm00487804_m1 | 0.94 | 0.0001 | 0.89 |
| nitric oxide synthase 2, inducible, macrophage | iNOS, Nos-2 | Mm00440485_m1 | 0.69 | 0.0416 | 0.47 |
| protein kinase C, mu | Prkcm, PKD1 | Mm00435790_m1 | 0.91 | 0.0006 | 0.84 |
| sialyltransferase 4C (beta-galactoside alpha-2,3-sialytransferase) | Siat4c,  ST3Gal IV | Mm00501503_m1 | 0.83 | 0.0052 | 0.69 |
| sialyltransferase 7 ((alpha-N-acetylneuraminyl 2,3-betagalactosyl-1,3)-N-acetyl galactosaminide alpha-2,6-sialyltransferase) D | Siat7d, ST6GalNAc IV | Mm00488228_m1 | 0.94 | 0.0001 | 0.89 |
| sialyltransferase 7 (alpha-N-acetylneuraminyl 2,3-beta-galactosyl-1,3)-N-acetyl galactosaminde alpha-2,6-sialyltransferase) A) | Siat7a, ST6GalNAc I | Mm00488218_m1 | 0.93 | 0.0003 | 0.87 |
| solute carrier family 10, member 2 | Slc10a2, ASBT | Mm00488258_m1 | 0.64 | 0.0661 | 0.40 |
| solute carrier family 15 (H+/peptide transporter), member 2 | Slc15a2, Pept2 | Mm00451610_m1 | 0.75 | 0.0212 | 0.56 |
| solute carrier family 26, member 3 | Slc26a3, Dra | Mm01291071_m1 | 1.00 | <0.0001 | 1.00 |
| solute carrier family 5 (iodide transporter), member 8 | Slc5a8, Ait | Mm00520629_m1 | 0.92 | 0.0005 | 0.84 |
| tumor necrosis factor | TNFa | [Mm00443258_m1](http://myscience.appliedbiosystems.com/servlet/com.celera.web.cdsentry.servlets.GetCdsEntryListServlet?cmd=cmdGetGeneExDetailReport&assayAcc=Mm00839397_m1) | 0.92 | 0.0004 | 0.85 |
