# Supplementary material for: Diarrhea as a cause of mortality in a mouse model of infectious colitis
Source: Genome Biol. 2008 Aug 4;9(8):R122. doi: 10.1186/gb-2008-9-8-r122 (PMC2575512; doi:10.1186/gb-2008-9-8-r122)
Supplement: Additional data file 4 — Side-by-side comparison of gene expression analyzed by microarray and qRT-PCR. [file gb-2008-9-8-r122-S4.doc]

**Additional data file 4.**  Side-by-side comparison of gene expression analyzed by microarray and qRT-PCR
