# Supplementary material for: Diarrhea as a cause of mortality in a mouse model of infectious colitis
Source: Genome Biol. 2008 Aug 4;9(8):R122. doi: 10.1186/gb-2008-9-8-r122 (PMC2575512; doi:10.1186/gb-2008-9-8-r122)
Supplement: Additional data file 6 — PCA parameters. [file gb-2008-9-8-r122-S6.doc]

| **Additional data file 6.** Two-dimensional principal component analysis (PCA) in the subspace of genes differentially regulated between FVB and SW mice. | | | |
| --- | --- | --- | --- |
|  | Sample | PC1  Infection status | PC2  Host genetic background |
| FVB uninfected controls (Fp) | F4_1 | 10.0345 | -24.7474 |
|  | F4_2 | 14.3277 | -24.3638 |
|  | F9_1 | 12.9357 | -23.798 |
|  | F9_2 | 9.91588 | -22.0406 |
|  | F9_3 | 6.89841 | -20.4539 |
| FVB 4 dpi (Fi4) | Fi4_1 | -6.26868 | -7.04468 |
|  | Fi4_2 | -10.0843 | -5.28929 |
|  | Fi4_3 | -6.18853 | -5.25129 |
| FVB 9 dpi (Fi9) | Fi9_1 | -40.8488 | -3.05197 |
|  | Fi9_2 | -48.3705 | -5.546 |
|  | Fi9_3 | -37.1858 | -2.71321 |
| SW uninfected controls (Sp) | S4_1 | 29.3196 | 7.28819 |
|  | S4_2 | 15.6219 | 3.93392 |
|  | S9_1 | 24.2379 | 2.07921 |
|  | S9_2 | 13.8939 | 5.7856 |
| SW 4 dpi (Si4) | SI4_1 | 4.58773 | 26.0865 |
|  | SI4_2 | 7.49687 | 22.5136 |
|  | SI4_3 | 9.4241 | 16.2882 |
| SW 9 dpi (Si9) | Si9_1 | -10.0365 | 20.8283 |
|  | Si9_2 | 5.91621 | 19.448 |
|  | Si9_3 | -5.62731 | 20.0485 |
