# Supplementary material for: Diarrhea as a cause of mortality in a mouse model of infectious colitis
Source: Genome Biol. 2008 Aug 4;9(8):R122. doi: 10.1186/gb-2008-9-8-r122 (PMC2575512; doi:10.1186/gb-2008-9-8-r122)
Supplement: Additional data file 7 — Hierarchical clustering of genes with host effect. [file gb-2008-9-8-r122-S7.doc]

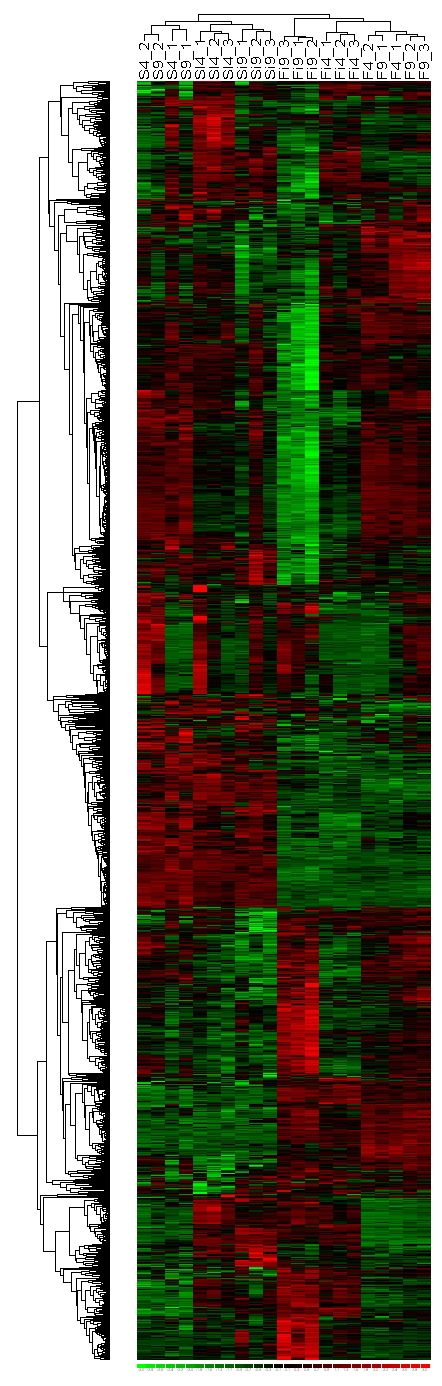


**Additional data file 7.** Hierarchical clustering of 1,547 genes exhibiting host effect.

Redundant probe sets were excluded from the analysis. The heat map shows color-coded expression levels (red = high expression, black = medium expression, and green = low expression). Gene trees are drawn vertically and sample trees vertically.
