# Supplementary material for: Diarrhea as a cause of mortality in a mouse model of infectious colitis
Source: Genome Biol. 2008 Aug 4;9(8):R122. doi: 10.1186/gb-2008-9-8-r122 (PMC2575512; doi:10.1186/gb-2008-9-8-r122)
Supplement: Additional data file 8 — The most significant variably expressed genes with host effect. [file gb-2008-9-8-r122-S8.doc]

| **Additional data file 8.** The most significant genes differentially expressed between susceptible and resistant mice prior to *C. rodentium* infection and/or at 4 and 9 dpi. | | | | | | | |
| --- | --- | --- | --- | --- | --- | --- | --- |
| Probe set ID | Gene | Gene name/  aliases | LocusLink | SW over FVB, control^a^ | SW over FVB,  4 dpi | SW over FVB,  9 dpi | Main functions |
| 1454714_x_at  1456471_x_at | 3-phosphoglycerate dehydrogenase | Phgdh | 236539 | -3.37*** | -5.2*** | -4.8** | Oxidoreductase activity, amino acid biosynthesis, metabolism |
| 1418979_at | aldo-keto reductase family 1, member C14 | Akr1c14 | 105387 |  |  | 3.06* | Oxidoreductase activity |
| 1417920_at | amnionless | Amn | 93835 | 3.58**** | 3.06**** | 1.9 | Development, protein localization |
| 1438364_x_at | angiogenin, ribonuclease A family, member 4 ^b^ | Ang4 | 219033 | 1.39 | 3.56 | -2.09 | Antimicrobial humoral response, defense response to bacteria |
| 1418069_at | apolipoprotein C-II | Apoc2 | 11813 | 0.53 |  | 3.61* | Lipid transporter activity |
| 1417828_at | **aquaporin 8 ^b^** | Aqp8 | 11833 |  |  | 3.14* | Water channel activity, transport |
| 1438841_s_at | arginase type II **^b^** | Arg2 | 11847 | 3.09** | 0.96 | 2.64* | Arginine metabolism |
| 1419393_at | ATP-binding cassette, sub-family G (WHITE), member 5 | Abcg5, sterolin 1 | 27409 | 1.02 | 0.64 | 3.18*** | ATPase activity, transport |
| 1455869_at | **calcium/calmodulin-dependent protein kinase II, beta** | Camk2b | 12323 | -0.85 | -3.39 | -2.46 | G1/S transition, calcium ion transport, calcium signaling |
| 1416193_at | **carbonic anhydrase 1 ^b^** | Car1, CA I | 12346 |  |  | 3.4* | one-carbon compound metabolism, maintenance of pH , anion transport |
| 1448949_at  1418094_s_at | **carbonic anhydrase 4 ^b^** | Car4, CA IV | 12351 |  |  | 5.32* | one-carbon compound metabolism, maintenance of pH , anion transport |
| 1416306_at | chloride channel calcium activated 3 | Clca3, gob-5 | 23844 |  | 0.65 | 3.06 | Chloride transport |
| 1418626_a_at  1454849_x_at | clusterin | Clu, ApoJ | 12759 |  | 0.71 | -3.17* | Cell death |
| 1419349_a_at | cytochrome P450, family 2, subfamily d, polypeptide 9 | Cyp2d9 | 13105 |  |  | 3.04* | Oxidoreductase activity |
| 1440016_at | dihydrolipoamide branched chain transacylase E2 | Dbt | 13171 | 3.63* | 2.52 | 1.37 | Metabolism |
| 1458099_at | EST |  |  | -3.21* |  | -1.43 |  |
| 1446934_at | EST |  |  | -3.49* | -2.36* | -2.73 |  |
| 1451948_at | gene model 1409, (NCBI) | Gm1409, IGKV5-39 | 620017 |  | 2.66* | 3.24* | Immunoglobulin-like |
| 1449526_a_at | glycerophosphodiester phosphodiesterase domain containing 3 | Gdpd3 | 68616 | -5.1**** | -4.35*** | -3.98** | Glycerol metabolism |
| 1457231_at | hypoxia inducible factor 1, alpha subunit | Hif1a, MOP1 | 15251 | 1.64 | 2.9* | 3.05** | Cell differentiation, signal transduction, transcription factor activity |
| 1460423_x_at | Ig kappa chain | Cr1, IgK | 381774 | -1.18 | 2.33* | 3.28** | Immune response |
| 1429381_x_at  1421653_a_at  1425763_x_at | immunoglobulin heavy chain (J558 family) | Igh-VJ558, Igh-A | 16061 | -2.12 | 1.15* | 3.65* | Immune response |
| 1460423_x_at | immunoglobulin heavy chain (V7183 family)  Ig kappa chain variable 1 (V1) Ig kappa chain variable 1-117  Ig kappa chain | Igh-V7183  Igk-V1  Igkv1-117  Cr1, IgK | 16059 16081 16098  381774 | -1.18 | 2.33* | 3.28** | Immune response |
| 1452577_at | immunoglobulin heavy chain complex | Igh, J558 | 111507 | 2.19 | 4.52* | 1.89* | Immune response |
| 1424931_s_at | immunoglobulin lambda chain, variable 1 ^b^ | Igl-V1 | 16142 | 0.83 | 4.08** | 1.28 | Antigen binding, humoral immune response |
| 1418165_at | intelectin a ^b^ | Itlna, Lfr | 16429 | 3.14* | 5.67* | -0.44 | Sugar binding, response to nematode |
| Probe set ID | Gene | Gene name/  aliases | LocusLink | SW over FVB, control^a^ | SW over FVB,  4 dpi | SW over FVB,  9 dpi | Main functions |
|  |  |  |  |  |  |  |  |
| 1457666_s_at | interferon activated gene 202b^b^ | Ifi202b | 26388 | -2.04 | -2.4 | -4.3** | Immune response |
| 1426906_at | interferon activated gene 203^b^ | Ifi203 | 15950 | 5.17** | 5.82* | 4.52** | Immune response, regulation of transcription |
| 1422071_at | lectin, galactose binding, soluble 6 | Lgals6, galectin-6 | 16857 | 3.61* |  | 2.05 | Sugar binding, immunomodulation |
| 1426573_at | malic enzyme 2, NAD(+)-dependent, mitochondrial | ME2,  NAD-ME | 107029 | 2.55**** | 2.32* | 3.08*** | Oxidoreductase activity, metabolism |
| 1423467_at | membrane-spanning 4-domains, subfamily A, member 4B | Ms4a4b, Chandra | 60361 | 2.12* | 3.15* | 3.0* | Immune response, Th1 differentiation |
| 1447252_s_at  1450719_at | meprin 1 alpha | Mep1a | 17287 | 0.71* | 0.9* | 3.39* | Metallopeptidase activity, proteolysis |
| 1418215_at | meprin 1 beta | Mep1b | 17288 |  |  | 3.14* | Metallopeptidase activity, proteolysis |
| 1453070_at | protocadherin 17 | Pcdh17 | 219228 | -3.24*** | -1.95 | -2.09 | Calcium ion binding |
| 1432579_at | radial spokehead-like 2 | Rshl2a | 66832 | 0.68 | 3.16*** | 0.97* | Transporter activity |
| 1418368_at | resistin like beta | Retnlb, Fizz2 | 57263 | -3.1 |  |  | Hormone activity |
| 1427963_s_at | retinol dehydrogenase 9 | Rdh9 | 103142 | 0.6 |  | 3.14** | Oxidoreductase activity, transport |
| 1424784_at | RIKEN cDNA 1700029I01 |  | 433791 | -2.75**** | -3.15** | -2.54 | Zinc ion binding |
| 1428359_s_at  1428358_at  1434137_x_at | RIKEN cDNA 1810010M01 | ZG16p | 69036 |  |  | 3.94 | Sugar binding |
| 1417735_at | RIKEN cDNA 1810030J14 |  | 66289 | 0.57 | 1.43 | 4.75** | similar to Serum amyloid P-component precursor (SAP) |
| 1434152_at | RIKEN cDNA 2210421G13 |  | 108956 | 1.53 | 3.72**** | 2.01 | Lipid binding |
| 1428604_at | RIKEN cDNA 2610305D13 |  | 112422 | 2.5**** | 3.17*** | 2.68* | Zinc ion binding, regulation of transcription |
| 1426936_at | RIKEN cDNA 2610305J24 |  | 192885 | 2.91** | 2.87*** | 4.18* |  |
| 1429896_at | RIKEN cDNA 5830408B19 |  | 74756 | -0.87* | -0.76** | -3.71* |  |
| 1437128_a_at | RIKEN cDNA A630033E08 |  | 240041 | -1.76 | -2.69** | -3.81*** | Zinc ion binding, regulation of transcription |
| 1460147_at | RIKEN cDNA A730009E18 |  | 319603 | -3.07**** | -2.59** | -2.59** |  |
| 1439065_x_at | Similar to zinc finger protein 665 |  | 626316 | 2.48* | 3.03* | 3.58*** | Zinc ion binding, regulation of transcription |
| 1417600_at | **solute carrier family 15 (H+/ peptide transporter), member 2 ^b^** | Slc15a2, Pept2 | 57738 | -5.28**** | -3.69** | -5.53**** | oligopeptide transport |
| 1419343_at | **solute carrier family 15 (oligopeptide transporter), member 1** | Slc15a1, Pept1 | 56643 |  | 0.59 | 3.79* | oligopeptide transport |
| 1429467_s_at  1421445_at  1427547_a_at | **solute carrier family 26, member 3 ^b^** | Slc26a3, Dra | 13487 |  |  | 6.04** | Anion exchanger activity, transport |
| 1444057_at | Transcribed locus |  |  | 3.03**** | 2.36* | 1.11 |  |
| 1456111_at | Transcribed locus |  |  | 2.41 | 4.09*** | 0.87 |  |
| 1459649_at | Transcribed locus |  |  | 4.08**** | 3.27** | 3.44* |  |
| 1451602_at | **sorting nexin 6** | Snx6, TFAF2 | 72183 | -3.98**** | -4.01 | -4.95** | Protein and ion transport |
| 1447458_at  1425668_a_at | ST3 beta-galactoside alpha-2,3-sialyltransferase 4, mRNA ^b^ | Siat4c, St3gal4 | 20443 | 6.24**** | 4.99**** | 5.65*** | Glycosyl transferase activity, protein amino acid glycosylation |
| 1421517_at | ST6 (alpha-N-acetyl-neuraminyl-2,3-beta-galactosyl-1,3)-N-acetylgalactosaminide alpha-2,6-sialyltransferase 1 ^b^ | Siat7a, St6galnac1 | 20445 | 2.42* | 3.29*** | 1.95* | Glycosyl transferase activity, protein amino acid glycosylation |
| 1450813_a_at | troponin I, skeletal, slow 1 | Tnni1 | 21952 |  |  | 3.24* | Actin binding, structural constituent of cytoskeleton |
| 1419453_at | ubiquitin carboxyl-terminal esterase L5 | Uchl5, Uch37 | 56207 | 3.22**** | 3.56*** | 3.23*** | ubiquitin-dependent protein catabolism |

^a^, the numbers represent log2 ratios resulted from individual groups comparison.

^b^, genes, which expression was confirmed by quantitative RT-PCR.

Significance by *t*-test was

*, p < 0.05

** p ≤ 0.005

***, p ≤ 0.0005

****, p ≤ 0.00005

Genes discussed in the paper are bolded
