# Supplementary material for: Diarrhea as a cause of mortality in a mouse model of infectious colitis
Source: Genome Biol. 2008 Aug 4;9(8):R122. doi: 10.1186/gb-2008-9-8-r122 (PMC2575512; doi:10.1186/gb-2008-9-8-r122)
Supplement: Additional data file 10 — Enrichment by GO categories of the most differentially expressed genes with host effect. [file gb-2008-9-8-r122-S10.doc]

| **Additional data file 10.** Enrichment of GO categories from analysis of the most differentially expressed genes exhibiting host effect  (p<0.05 by Hypergeometric test and at least 4 functionally annotated genes) | | |
| --- | --- | --- |
|  |  |  |
| **probe set** | **Gene** | **LocusLink** |
|  |  |  |
| **Found 9 Gene Ontology "transporter activity" genes in a list with 48 annotated genes (all: 2752/31850, PValue: 0.020300) **** | | |
| 1416306_at | chloride channel calcium activated 3 | 23844 |
| 1417600_at | solute carrier family 15 (H+/peptide transporter), member 2 | 57738 |
| 1417828_at | aquaporin 8 | 11833 |
| 1418069_at | apolipoprotein C-II | 11813 |
| 1419343_at | solute carrier family 15 (oligopeptide transporter), member 1 | 56643 |
| 1419393_at | ATP-binding cassette, sub-family G (WHITE), member 5 | 27409 |
| 1421445_at | solute carrier family 26, member 3 | 13487 |
| 1451602_at | sorting nexin 6 | 72183 |
| 1432579_at | radial spokehead-like 2 | 66832 |
|  |  |  |
| **Found 5 Gene Ontology "carrier activity" genes in a list with 48 annotated genes (all: 761/31850, PValue: 0.005634) ***** | | |
| 1416306_at | chloride channel calcium activated 3 | 23844 |
| 1417600_at | solute carrier family 15 (H+/peptide transporter), member 2 | 57738 |
| 1419343_at | solute carrier family 15 (oligopeptide transporter), member 1 | 56643 |
| 1421445_at | solute carrier family 26, member 3 | 13487 |
| 1432579_at | radial spokehead-like 2 | 66832 |
|  |  |  |
| **Found 12 Gene Ontology "extracellular region" genes in a list with 48 annotated genes (all: 3950/31850, PValue: 0.012438) **** | | |
| 1417735_at | RIKEN cDNA 1810030J14 gene /// similar to Serum amyloid P-component precursor (SAP) | 630754 /// 66289 |
| 1417920_at | amnionless | 93835 |
| 1418069_at | apolipoprotein C-II | 11813 |
| 1418094_s_at | carbonic anhydrase 4 | 12351 |
| 1418165_at | intelectin a | 16429 |
| 1418215_at | meprin 1 beta | 17288 |
| 1418368_at | resistin like beta | 57263 |
| 1418626_a_at | clusterin | 12759 |
| 1425668_a_at | ST3 beta-galactoside alpha-2,3-sialyltransferase 4 | 20443 |
| 1450719_at | meprin 1 alpha | 17287 |
| 1434152_at | RIKEN cDNA 2210421G13 gene /// hypothetical protein LOC193676 /// similar to apolipoprotein L, 3 (predicted) | 108956 /// 193676 /// 666661 |
| 1438364_x_at | angiogenin, ribonuclease A family, member 4 | 219033 |
|  |  |  |
| **Found 11 Gene Ontology "extracellular space" genes in a list with 48 annotated genes (all: 3531/31850, PValue: 0.014240) **** | | |
| 1417735_at | RIKEN cDNA 1810030J14 gene /// similar to Serum amyloid P-component precursor (SAP) | 630754 /// 66289 |
| 1417920_at | amnionless | 93835 |
| 1418069_at | apolipoprotein C-II | 11813 |
| 1418094_s_at | carbonic anhydrase 4 | 12351 |
| 1418165_at | intelectin a | 16429 |
| 1418215_at | meprin 1 beta | 17288 |
| 1418368_at | resistin like beta | 57263 |
| 1418626_a_at | clusterin | 12759 |
| 1425668_a_at | ST3 beta-galactoside alpha-2,3-sialyltransferase 4 | 20443 |
| 1450719_at | meprin 1 alpha | 17287 |
| 1438364_x_at | angiogenin, ribonuclease A family, member 4 | 219033 |
|  |  |  |

| **Additional data file 10.** Continued | | |
| --- | --- | --- |
|  |  |  |
| **probe set** | **gene** | **LocusLink** |
|  |  |  |
| **Found 4 Gene Ontology "immune response" genes in a list with 48 annotated genes (all: 667/31850, PValue: 0.017922) **** | | |
| 1421551_s_at | interferon activated gene 202B | 26388 |
| 1424931_s_at | immunoglobulin lambda chain, variable 1 | 16142 |
| 1426906_at | interferon activated gene 203 | 15950 |
| 1438364_x_at | angiogenin, ribonuclease A family, member 4 | 219033 |
|  |  |  |
| **Found 6 Gene Ontology "oxidoreductase activity" genes in a list with 48 annotated genes (all: 1491/31850, PValue: 0.023848) **** | | |
| 1418979_at | aldo-keto reductase family 1, member C14 | 105387 |
| 1419349_a_at | cytochrome P450, family 2, subfamily d, polypeptide 9 | 13105 |
| 1426573_at | malic enzyme 2, NAD(+)-dependent, mitochondrial | 107029 |
| 1427963_s_at | retinol dehydrogenase 9 | 103142 |
| 1454714_x_at | 3-phosphoglycerate dehydrogenase /// similar to 3-phosphoglycerate dehydrogenase /// similar to 3-phosphoglycerate dehydrogenase /// similar to 3-phosphoglycerate dehydrogenase /// similar to 3-phosphoglycerate dehydrogenase | 236539 /// 668771 /// 670155 /// 671972 /// 673015 |
| 1457231_at | Hypoxia inducible factor 1, alpha subunit | 15251 |
|  |  |  |
| **Found 11 Gene Ontology "extracellular region part" genes in a list with 48 annotated genes (all: 3743/31850, PValue: 0.021262) **** | | |
| 1417735_at | RIKEN cDNA 1810030J14 gene /// similar to Serum amyloid P-component precursor (SAP) | 630754 /// 66289 |
| 1417920_at | amnionless | 93835 |
| 1418069_at | apolipoprotein C-II | 11813 |
| 1418094_s_at | carbonic anhydrase 4 | 12351 |
| 1418165_at | intelectin a | 16429 |
| 1418215_at | meprin 1 beta | 17288 |
| 1418368_at | resistin like beta | 57263 |
| 1418626_a_at | clusterin | 12759 |
| 1425668_a_at | ST3 beta-galactoside alpha-2,3-sialyltransferase 4 | 20443 |
| 1450719_at | meprin 1 alpha | 17287 |
| 1438364_x_at | angiogenin, ribonuclease A family, member 4 | 219033 |
|  |  |  |
| **Found 4 Protein Domain "Immunoglobulin-like // 3.4E-7" genes in a list with 18 annotated genes (all: 326/10020, PValue: 0.002344) ***** | | |
| 1421653_a_at | immunoglobulin heavy chain (J558 family) /// similar to immunoglobulin heavy chain variable region /// similar to immunoglobulin mu-chain /// similar to anti-poly(dC) monoclonal antibody heavy chain | 16061 /// 238447 /// 544903 /// 544907 |
| 1451948_at | gene model 1409, (NCBI) | 620017 |
| 1452577_at | Immunoglobulin heavy chain complex | 111507 |
| 1460423_x_at | immunoglobulin heavy chain (V7183 family) /// immunoglobulin kappa chain variable 1 (V1) /// immunoglobulin kappa chain variable 1-117 /// Ig kappa chain | 16059 /// 16081 /// 16098 /// 381774 |
|  |  |  |
| **Found 8 Chromosome "12" genes in a list with 49 annotated genes (all: 1367/37379, PValue: 0.000372) ****** | | |
| 1417920_at | amnionless | 93835 |
| 1421445_at | solute carrier family 26, member 3 | 13487 |
| 1421653_a_at | immunoglobulin heavy chain (J558 family) /// similar to immunoglobulin heavy chain variable region /// similar to immunoglobulin mu-chain /// similar to anti-poly(dC) monoclonal antibody heavy chain | 16061 /// 238447 /// 544903 /// 544907 |
| 1438841_s_at | arginase type II | 11847 |
| 1451602_at | sorting nexin 6 | 72183 |
| 1452577_at | Immunoglobulin heavy chain complex | 111507 |
| 1460423_x_at | immunoglobulin heavy chain (V7183 family) /// immunoglobulin kappa chain variable 1 (V1) /// immunoglobulin kappa chain variable 1-117 /// Ig kappa chain | 16059 /// 16081 /// 16098 /// 381774 |
| 1457231_at | Hypoxia inducible factor 1, alpha subunit | 15251 |
|  |  |  |
