# Supplementary material for: Diarrhea as a cause of mortality in a mouse model of infectious colitis
Source: Genome Biol. 2008 Aug 4;9(8):R122. doi: 10.1186/gb-2008-9-8-r122 (PMC2575512; doi:10.1186/gb-2008-9-8-r122)
Supplement: Additional data file 13 — Enrichment by GO categories of the most differentially expressed genes from delta eta analysis. [file gb-2008-9-8-r122-S13.doc]

| **Additional data file 13.** Enrichment of GO categories of the most differentially expressed genes from delta eta analysis (p<0.05 by Hypergeometric test and at least 4 functionally annotated genes) | | |
| --- | --- | --- |
|  | |  |
| **gene** | | **LocusLink** |
|  | |  |
| **Found 7 "transporter activity" genes in a list with 30 annotated genes (all: 2752/31850, PValue: 0.012322) **** | | |
| 1416306_at | chloride channel calcium activated 3 | 23844 |
| 1417828_at | aquaporin 8 | 11833 |
| 1418069_at | apolipoprotein C-II | 11813 |
| 1419343_at | solute carrier family 15 (oligopeptide transporter), member 1 | 56643 |
| 1425382_a_at | aquaporin 4 | 11829 |
| 1427547_a_at | solute carrier family 26, member 3 | 13487 |
| 1439727_at | chloride channel calcium activated 6 /// expressed sequence AI747448 | 99663 /// 99709 |
|  |  |  |
| **Found 6 "immune system process" genes in a list with 30 annotated genes (all: 1101/31850, PValue: 0.000490) ****** | | |
| **Found 6 "immune response" genes in a list with 30 annotated genes (all: 667/31850, PValue: 0.000032) ******* | | |
| **Found 6 "humoral immune response" genes in a list with 30 annotated genes (all: 116/31850, PValue: 0.000000) *********** | | |
| **Found 6 "physiological response to stimulus" genes in a list with 30 annotated genes (all: 1417/31850, PValue: 0.001816) ***** | | |
| 1423153_x_at | complement component factor h | 12628 |
| 1424305_at | immunoglobulin joining chain | 16069 |
| 1424931_s_at | immunoglobulin lambda chain, variable 1 | 16142 |
| 1427660_x_at | immunoglobulin kappa chain variable 28 (V28) /// immunoglobulin kappa chain variable 8 (V8)-16 | 16114 /// 640340 |
| 1427860_at | immunoglobulin kappa chain variable 6-23 /// immunoglobulin kappa chain variable 19 (V19)-14 /// immunoglobulin kappa chain variable 19 (V19)-13 /// similar to Ig kappa chain V-V region MPC11 precursor /// similar to Ig kappa chain V-V region MPC11 precursor /// similar to Ig kappa chain V-V region MPC11 precursor /// similar to Ig kappa chain V-V region MPC11 precursor /// similar to Ig kappa chain V-V region MPC11 precursor | 637227 /// 667881 /// 667899 /// 669053 /// 669070 /// 676136 /// 676162 /// 676175 |
| 1438364_x_at | angiogenin, ribonuclease A family, member 4 | 219033 |
|  |  |  |
| **Found 6 "antigen binding" genes in a list with 30 annotated genes (all: 103/31850, PValue: 0.000000) *********** | | |
| 1424305_at | immunoglobulin joining chain | 16069 |
| 1424931_s_at | immunoglobulin lambda chain, variable 1 | 16142 |
| 1425763_x_at | immunoglobulin heavy chain (J558 family) /// similar to immunoglobulin heavy chain variable region /// similar to immunoglobulin mu-chain /// similar to anti-poly(dC) monoclonal antibody heavy chain | 16061 /// 238447 /// 544903 /// 544907 |
| 1427660_x_at | immunoglobulin kappa chain variable 28 (V28) /// immunoglobulin kappa chain variable 8 (V8)-16 | 16114 /// 640340 |
| 1427860_at | immunoglobulin kappa chain variable 6-23 /// immunoglobulin kappa chain variable 19 (V19)-14 /// immunoglobulin kappa chain variable 19 (V19)-13 /// similar to Ig kappa chain V-V region MPC11 precursor /// similar to Ig kappa chain V-V region MPC11 precursor /// similar to Ig kappa chain V-V region MPC11 precursor /// similar to Ig kappa chain V-V region MPC11 precursor /// similar to Ig kappa chain V-V region MPC11 precursor | 637227 /// 667881 /// 667899 /// 669053 /// 669070 /// 676136 /// 676162 /// 676175 |
| 1452463_x_at | Immunoglobulin kappa chain complex | 243469 |
|  |  |  |
| **Found 4 "sugar binding" genes in a list with 30 annotated genes (all: 337/31850, PValue: 0.000272) ****** | | |
| **Found 4 "carbohydrate binding" genes in a list with 30 annotated genes (all: 500/31850, PValue: 0.001190) ***** | | |
| 1418165_at | intelectin a | 16429 |
| 1422071_at | lectin, galactose binding, soluble 6 | 16857 |
| 1434137_x_at | RIKEN cDNA 1810010M01 gene | 69036 |
| 1451440_at | chondrolectin | 246048 |
|  |  |  |
|  | |  |
|  |  |  |
| **Additional data file 13.** Continued | | |
|  | |  |
| **gene** | | **LocusLink** |
|  | |  |
| **Found 11 "extracellular region" genes in a list with 30 annotated genes (all: 3950/31850, PValue: 0.000597) ****** | | |
| **Found 11 "extracellular space" genes in a list with 30 annotated genes (all: 3531/31850, PValue: 0.000224) ****** | | |
| **Found 11 "extracellular region part" genes in a list with 30 annotated genes (all: 3743/31850, PValue: 0.000374) ****** | | |
| 1417735_at | RIKEN cDNA 1810030J14 gene /// similar to Serum amyloid P-component precursor (SAP) | 630754 /// 66289 |
| 1418069_at | apolipoprotein C-II | 11813 |
| 1418094_s_at | carbonic anhydrase 4 | 12351 |
| 1418165_at | intelectin a | 16429 |
| 1418215_at | meprin 1 beta | 17288 |
| 1418368_at | resistin like beta | 57263 |
| 1419476_at | ADAM-like, decysin 1 | 58860 |
| 1424305_at | immunoglobulin joining chain | 16069 |
| 1449033_at | tumor necrosis factor receptor superfamily, member 11b (osteoprotegerin) | 18383 |
| 1438364_x_at | angiogenin, ribonuclease A family, member 4 | 219033 |
| 1439853_at | beta-1,4-N-acetyl-galactosaminyl transferase 2 | 14422 |
|  |  |  |
| **Found 4 "channel or pore class transporter activity" genes in a list with 30 annotated genes (all: 777/31850, PValue: 0.005817) ***** | | |
| **Found 4 "alpha-type channel activity" genes in a list with 30 annotated genes (all: 732/31850, PValue: 0.004716) ***** | | |
| 1416306_at | chloride channel calcium activated 3 | 23844 |
| 1417828_at | aquaporin 8 | 11833 |
| 1425382_a_at | aquaporin 4 | 11829 |
| 1439727_at | chloride channel calcium activated 6 /// expressed sequence AI747448 | 99663 /// 99709 |
|  |  |  |
| **Found 8 "organismal physiological process" genes in a list with 30 annotated genes (all: 2672/31850, PValue: 0.002650) ***** | | |
| 1423153_x_at | complement component factor h | 12628 |
| 1424305_at | immunoglobulin joining chain | 16069 |
| 1424931_s_at | immunoglobulin lambda chain, variable 1 | 16142 |
| 1425382_a_at | aquaporin 4 | 11829 |
| 1427660_x_at | immunoglobulin kappa chain variable 28 (V28) /// immunoglobulin kappa chain variable 8 (V8)-16 | 16114 /// 640340 |
| 1427860_at | immunoglobulin kappa chain variable 6-23 /// immunoglobulin kappa chain variable 19 (V19)-14 /// immunoglobulin kappa chain variable 19 (V19)-13 /// similar to Ig kappa chain V-V region MPC11 precursor /// similar to Ig kappa chain V-V region MPC11 precursor /// similar to Ig kappa chain V-V region MPC11 precursor /// similar to Ig kappa chain V-V region MPC11 precursor /// similar to Ig kappa chain V-V region MPC11 precursor | 637227 /// 667881 /// 667899 /// 669053 /// 669070 /// 676136 /// 676162 /// 676175 |
| 1450813_a_at | troponin I, skeletal, slow 1 | 21952 |
| 1438364_x_at | angiogenin, ribonuclease A family, member 4 | 219033 |
|  |  |  |
