# Supplementary material for: Diarrhea as a cause of mortality in a mouse model of infectious colitis
Source: Genome Biol. 2008 Aug 4;9(8):R122. doi: 10.1186/gb-2008-9-8-r122 (PMC2575512; doi:10.1186/gb-2008-9-8-r122)
Supplement: Additional data file 16 — FatiGO analysis on genes identified by BioConductor analysis. [file gb-2008-9-8-r122-S16.doc]

**
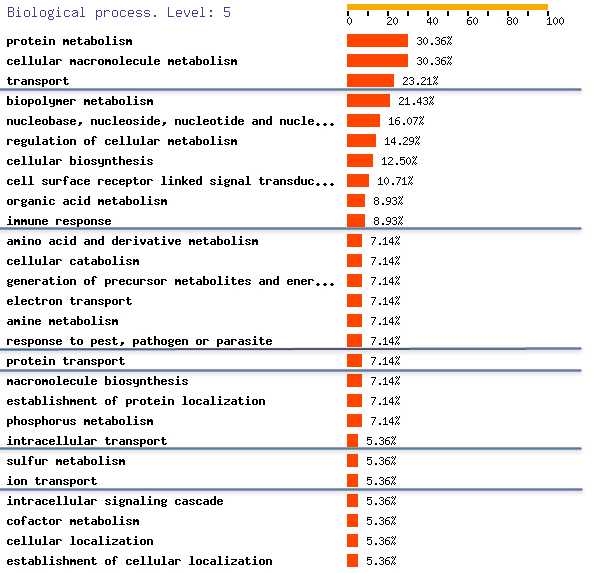
**

**A.**

**
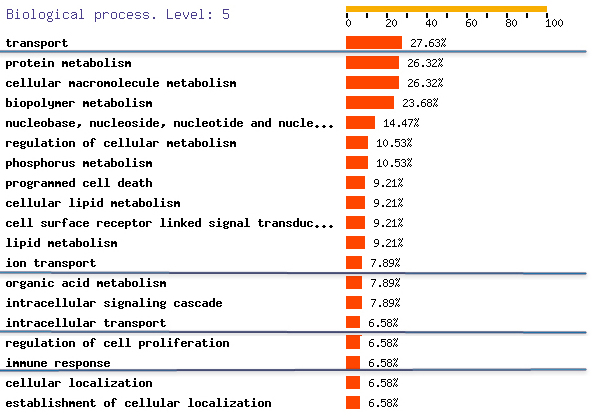
**

**B.**

**Additional data file 16.** FatiGO (fast assignment and transference of information) analysis of genes with host effect (**A**) and infection x host effect (**B**) identified by BioConductor analysis. After exclusion of unknown and redundant probe sets, 106 out of 232 genes and 126 out of 167 genes were analyzed respectively. Transport-related categories were markedly overrepresented when compared with immune-related ones, recapitulating the results obtained by dChip analysis.
