# Supplementary material for: Diarrhea as a cause of mortality in a mouse model of infectious colitis
Source: Genome Biol. 2008 Aug 4;9(8):R122. doi: 10.1186/gb-2008-9-8-r122 (PMC2575512; doi:10.1186/gb-2008-9-8-r122)
Supplement: Additional data file 17 — Enrichment by GO categories of genes detected by BioConductor analysis. [file gb-2008-9-8-r122-S17.doc]

| **Additional data file 17.** Enrichment of genes detected by BioConductor analysis by Hypergeometric test with p<0.0005 |
| --- |
|  |
| **Host effect -** Found 232 genes or SNPs; 21 redundant probe sets excluded; 211 left |
|  |
| Finding significant Gene Ontology clusters... |
| Found 4 "phosphoric ester hydrolase activity" genes in a cluster with 17 annotated genes (all: 687/31850, PValue: 0.000408) |
| Found 5 "hydrolase activity\, acting on ester bonds" genes in a cluster with 16 annotated genes (all: 1367/31850, PValue: 0.000425) |
|  |
| Finding significant Chromosome clusters... |
| Found 5 "2 H4" genes in a cluster with 175 annotated genes (all: 118/37379, PValue: 0.000242) |
| Found 6 "4 E1" genes in a cluster with 175 annotated genes (all: 116/37379, PValue: 0.000019) |
|  |
| **Infection effect -** Found 167 genes or SNPs; 20 redundant probe sets excluded; 147 left |
|  |
| Finding significant Gene Ontology clusters... |
| Found 48 "membrane" genes in a cluster with 90 annotated genes (all: 10764/31850, PValue: 0.000106) |
| Found 39 "membrane part" genes in a cluster with 90 annotated genes (all: 8454/31850, PValue: 0.000419) |
| Found 5 "lytic vacuole" genes in a cluster with 79 annotated genes (all: 258/31850, PValue: 0.000464) |
| Found 17 "transporter activity" genes in a cluster with 79 annotated genes (all: 2752/31850, PValue: 0.000353) |
| | 1417828_at | aquaporin 8 | | --- | --- | | 1422699_at | arachidonate 12-lipoxygenase | | 1421129_a_at | ATPase, Ca++ transporting, ubiquitous | | 1428988_at | ATP-binding cassette, sub-family C (CFTR/MRP), member 3 | | 1435613_x_at | cytochrome c oxidase, subunit Vb | | 1425525_a_at | purinergic receptor P2X, ligand-gated ion channel 4 | | 1424364_a_at | RIKEN cDNA 1110020P15 gene | | 1452326_at | RIKEN cDNA 1810010G06 gene | | 1419343_at | solute carrier family 15 (oligopeptide transporter), member 1 | | 1426600_at | solute carrier family 2 (facilitated glucose transporter), member 1 | | 1421445_at | solute carrier family 26, member 3 | | 1428793_at | solute carrier family 36 (proton/amino acid symporter), member 1 | | 1452445_at | solute carrier family 41, member 2 | | 1425606_at | solute carrier family 5 (iodide transporter), member 8 | | 1426069_s_at | solute carrier family 7 (cationic amino acid transporter, y+ system), member 4 | | 1437259_at | solute carrier family 9 (sodium/hydrogen exchanger), member 2 | | 1429240_at | StAR-related lipid transfer (START) domain containing 4 | | 1441927_at | Synaptotagmin VII | |
| Found 5 "lysosome" genes in a cluster with 79 annotated genes (all: 258/31850, PValue: 0.000464) |
| Found 31 "integral to membrane" genes in a cluster with 70 annotated genes (all: 7545/31850, PValue: 0.000119) |
| Found 31 "intrinsic to membrane" genes in a cluster with 70 annotated genes (all: 7564/31850, PValue: 0.000125) |
| Found 5 "vacuole" genes in a cluster with 68 annotated genes (all: 301/31850, PValue: 0.000467) |
| Found 5 "integral to plasma membrane" genes in a cluster with 18 annotated genes (all: 1199/31850, PValue: 0.000426) |
| Found 5 "intrinsic to plasma membrane" genes in a cluster with 18 annotated genes (all: 1220/31850, PValue: 0.000462) |
|  |
| Finding significant Chromosome clusters... |
| Found 5 "10 C1" genes in a cluster with 93 annotated genes (all: 254/37379, PValue: 0.000445) |
|  |
