# Supplementary material for: Diarrhea as a cause of mortality in a mouse model of infectious colitis
Source: Genome Biol. 2008 Aug 4;9(8):R122. doi: 10.1186/gb-2008-9-8-r122 (PMC2575512; doi:10.1186/gb-2008-9-8-r122)
Supplement: Additional data file 18 — Genes with most predictive power obtained from BioConductor analysis. [file gb-2008-9-8-r122-S18.doc]

**
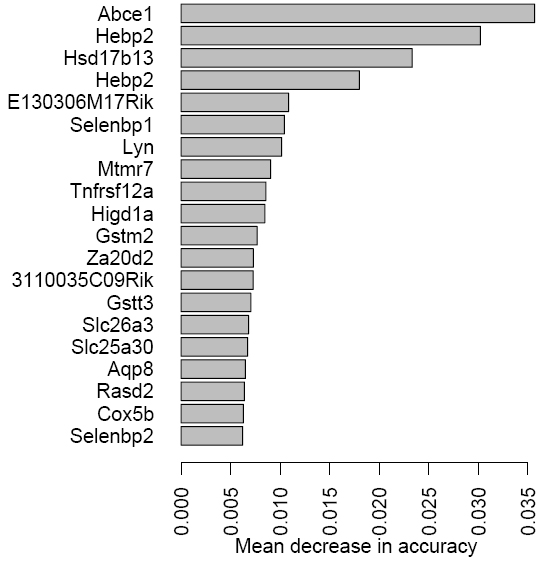
**

**Additional data file 18.** Genes with most predictive power obtained from BioConductor analysis.

When random forest evaluation of gene importance in predicting four classes (host x infection status) was applied, 19 genes appeared to have high predictive capacity (p<0.05). Genes such as *Slc26a3* and *Aqp8* were identified as most differentially expressed by dChip analysis as well.
