# Supplementary material for: Diarrhea as a cause of mortality in a mouse model of infectious colitis
Source: Genome Biol. 2008 Aug 4;9(8):R122. doi: 10.1186/gb-2008-9-8-r122 (PMC2575512; doi:10.1186/gb-2008-9-8-r122)
Supplement: Additional data file 19 — Hierarchical clustering of genes discussed in text as potentially contributing to development of intestinal ion disturbances and diarrhea. [file gb-2008-9-8-r122-S19.doc]

**
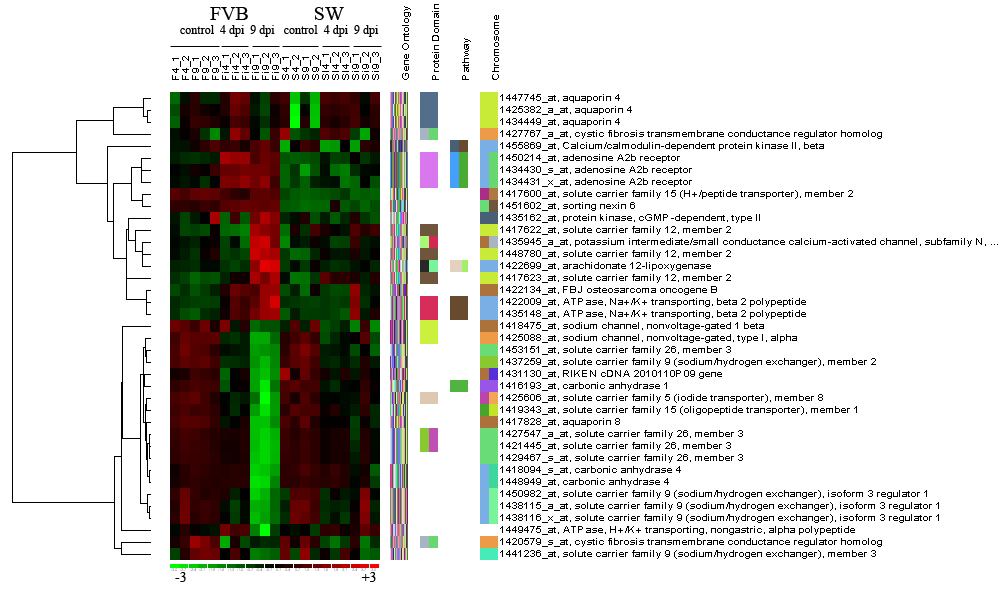
**

**Additional data file 19.** Hierarchical clustering of genes discussed in text as potentially contributing to development of intestinal ion disturbances and diarrhea.

Red and green colors indicate up- and downregulation respectively.
