# Supplementary material for: Diarrhea as a cause of mortality in a mouse model of infectious colitis
Source: Genome Biol. 2008 Aug 4;9(8):R122. doi: 10.1186/gb-2008-9-8-r122 (PMC2575512; doi:10.1186/gb-2008-9-8-r122)
Supplement: Additional data file 21 — Correlation of raw intensities between biological replicates validating microarray results. [file gb-2008-9-8-r122-S21.doc]

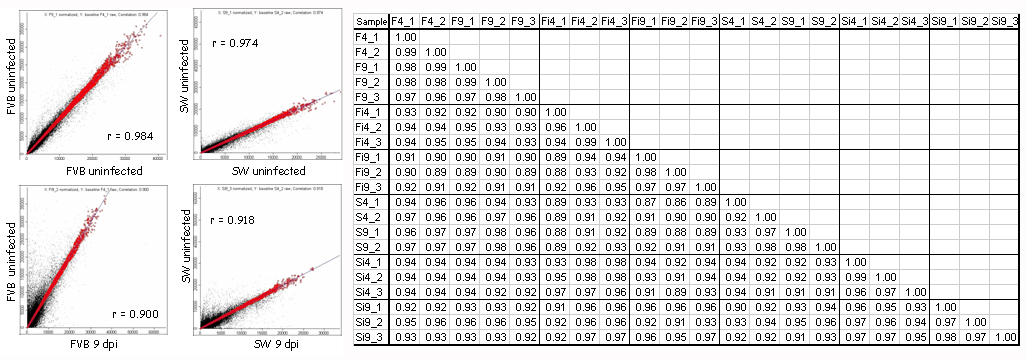


**Additional data file 21.** Correlation of raw intensities between biological replicates.

Microarray results were tightly correlated between biological replicates within FVB mice groups (r between 0.96 to 0.99 for controls, 0.94 to 0.99 for 4 dpi, and 0.97 to 0.98 for 9 dpi). Samples from uninoculated SW mice were less tightly correlated in accordance with the outbred nature of the animals (r between 0.92 to 0.98 for controls, 0.96 to 0.99 for 4 dpi, and 0.97 to 0.98 for 9 dpi). The correlations between the control and infected mice were smaller and reflected the severity of the disease progression. Thus, comparison of 4 dpi and 9 dpi FVB mice with the uninoculated controls or between each other resulted in r between 0.90 to 0.95, 0.89 to 0.92, and 0.88 to 0.96 respectively. Similar comparisons in SW mice lead to higher correlations (r between 0.91 to 0. 94, 0.90 to 0.96, and 0.93 to 0. 97 for 4 dpi and 9 dpi mice compared with controls or between each other) in accordance with the subclinical disease in the outbred animals. None of the correlations was substantially low (all r were higher than 0.85) indicating that 9 days of infection did not robustly change global gene expression profile.

1. Representative examples of array-to-array correlations between uninoculated and infected animals within the same genetic group.
2. Correlation matrix with r values for all array-to-array comparisons.

The following code for individual samples was used: F or S correspond to FVB or SW lines; “i” indicates infection status, 4 and 9 represent 4 and 9 dpi respectively, and “_1”, “_2”, and “_3” specify biological replicate.
